# Torsional force microscopy of van der Waals moirés and atomic lattices

**DOI:** 10.1073/pnas.2314083121

**Published:** 2024-03-01

**Authors:** Mihir Pendharkar, Steven J. Tran, Gregory Zaborski, Joe Finney, Aaron L. Sharpe, Rupini V. Kamat, Sandesh S. Kalantre, Marisa Hocking, Nathan J. Bittner, Kenji Watanabe, Takashi Taniguchi, Bede Pittenger, Christina J. Newcomb, Marc A. Kastner, Andrew J. Mannix, David Goldhaber-Gordon

**Affiliations:** ^a^Stanford Institute for Materials and Energy Sciences, SLAC National Accelerator Laboratory, Menlo Park, CA 94025; ^b^Department of Materials Science and Engineering, Stanford University, Stanford, CA 94305; ^c^Department of Physics, Stanford University, Stanford, CA 94305; ^d^Materials Physics Department, Sandia National Laboratories, Livermore, CA 94550; ^e^Independent Researcher, Palo Alto, CA 94305; ^f^Research Center for Electronic and Optical Materials, National Institute for Materials Science, Tsukuba 305-0044, Japan; ^g^Research Center for Materials Nanoarchitectonics, National Institute for Materials Science, Tsukuba 305-0044, Japan; ^h^Bruker Nano Surfaces, AFM Unit, Santa Barbara, CA 93117; ^i^Stanford Nano Shared Facilities, Stanford University, Stanford, CA 94305; ^j^Department of Physics, Massachusetts Institute of Technology, Cambridge, MA 02139

**Keywords:** moiré superlattices, dynamic friction, AFM techniques, imaging graphene heterostructures, van der Waals materials

## Abstract

In a stack of atomically thin van der Waals layers, introducing interlayer twist creates a moiré superlattice whose period is a function of twist angle. Changes in that twist angle of even hundredths of a degree can dramatically transform the system’s electronic properties. Setting a precise and uniform twist angle for a stack remains difficult; hence, determining that twist angle and mapping its spatial variation is very important. Techniques have emerged to do this by imaging the moiré, but most of these require sophisticated infrastructure, time-consuming sample preparation beyond stack synthesis, or both. In this work, we show that torsional force microscopy (TFM), a scanning probe technique sensitive to dynamic friction, can reveal surface and shallow subsurface structure of van der Waals stacks on multiple length scales: the moirés formed between bi-layers of graphene and between graphene and hexagonal boron nitride (hBN) and also the atomic crystal lattices of graphene and hBN. In TFM, torsional motion of an Atomic Force Microscope (AFM) cantilever is monitored as it is actively driven at a torsional resonance while a feedback loop maintains contact at a set force with the sample surface. TFM works at room temperature in air, with no need for an electrical bias between the tip and the sample, making it applicable to a wide array of samples. It should enable determination of precise structural information including twist angles and strain in moiré superlattices and crystallographic orientation of van der Waals flakes to support predictable moiré heterostructure fabrication.

## Significance

Moiré superlattices, formed by superimposing two layers of graphene or other two-dimensional (2D) materials at a relative twist angle, have been studied extensively, but techniques to directly image the moirés at room temperature and in air remain scarce. Here, we introduce torsional force microscopy (TFM), an AFM-based technique, that reveals moirés formed in atomically thin materials. We find that TFM also reveals atomic crystal lattices of these materials. TFM can be used to image 2D materials on polymer stamps, without the need for any electrical contacts, providing an unprecedented preview of structural properties of moiré heterostructures before, during, and after stacking.

The theoretical prediction of electronic Bloch bands in moiré superlattices in twisted van der Waals (VdW) bilayers (1–5) and the subsequent observations of a correlated insulator state and unconventional superconductivity in magic-angle twisted bilayer graphene (tBG) (6, 7) have unlocked a powerful new approach to tuning and discovering electronic properties of materials. tBG has displayed topological effects orbital ferromagnetism (8) and quantized anomalous hall effect (9), ferroelectricity (10), strange-metal behavior (11, 12), and more depending on interlayer twist angle, applied electric and magnetic fields, and other subtle structural features. For example, orbital ferromagnetism in tBG appears to depend on not only the twist between the two layers of graphene but also the twist between graphene and encapsulating hexagonal boron nitride (hBN) (13). Uniaxial strain has recently been found to dramatically influence electronic properties of tBG away from magic angle (14, 15). Beyond tBG, a burgeoning array of moiré systems, extending to more layers and different constituent layers, also show exciting behaviors. Unfortunately, moiré superlattices based on 2D materials are plagued by poor control, reproducibility, and spatial uniformity of twist angle and other structural properties (16). Convenient, rapid, and reliable techniques for imaging moiré superlattices will be needed to provide feedback to guide improvements in heterostructure synthesis.

Priorities for capabilities of such a technique should include 1) imaging moiré superlattices on the scale of individual unit cells (ranging from nanometers to microns), 2) imaging over large areas (microns), 3) imaging subsurface moiré superlattices, and 4) imaging atomic crystal lattices of VdW materials (sub-nanometer). This covers many but not all structural properties known to strongly influence electronic properties. As has been succinctly summarized by McGilly et al. (17), and is still true, techniques that depend on cryogenics, ultra-high vacuum, complex infrastructure, restrictive environmental controls, and/or extensive sample preprocessing (including nanofabrication) can provide powerful information but are not appropriate for quick feedback to stack synthesis. Instead, we should seek a technique that is “straightforward”: operating in air, at room temperature. To allow characterizing partially complete stacks, the technique should not require electrical contacts or modifications to the sample or its surface and should work on VdW stacks on soft polymers commonly used as stamps for stack assembly. Here, we aim to address the need for such a rapid feedback technique.

Multiple scanning probe techniques have recently been shown to provide structural information on moirés. Among those, some can be used in air at room temperature, often on a commercial AFM platform, offering the promise of tight feedback for heterostructure synthesis. Conductive AFM (C-AFM) can image atomic lattices (18), provided an electrical contact is made to a conductive sample or a conductive substrate below an atomically thin insulating sample. Simple tapping-mode AFM can image open-face graphene-hBN moirés and few-nanometer-deep hBN–hBN moirés with remarkable few-nanometer lateral resolution over microns (19). To our knowledge, this approach has not yet worked for tBG, nor has atomic-scale imaging been shown in ambient on atomically thin stacks. Scanning microwave impedance microscopy (s-MIM) has imaged open-face tBG moirés under ambient conditions (20, 21). Although it does not require an electrical sample contact, it does require specialized hardware and has not been shown to resolve atomic lattices. Lateral (or friction) force microscopy (LFM/FFM), a variant of contact AFM focusing on lateral rather than vertical tip deflection, has perhaps come the closest to providing a facile method for mapping structural features at both moiré scale (22, 23) and atomic lattice scale on hBN and graphite (24, 25): Evidently lateral friction forces vary with tiny changes in the positioning of the tip on the sample. Force modulation microscopy (FMM) and contact resonance AFM (CR-AFM) map topography by contact AFM while also driving the cantilever at or below a vertical (diving board) resonance of the cantilever to image the local stiffness of the surface. FMM too has been shown to image both moirés and atomic lattices (26). Both LFM and FMM satisfy most of the criteria laid out above but have not been shown to resolve subsurface moirés, to our knowledge.

Piezoresponse force microscopy (PFM), a contact-AFM technique, has produced remarkable maps of moirés with few-nanometer resolution over hundreds of nanometers (17). By superimposing two orthogonal scans, taken by rotating the sample by 90^°^, the full hexagonal unit cell of a tBG moiré has been imaged with lateral-PFM (L-PFM). Subsurface moirés were also observed, though atomic lattices have not been. The authors shared their surprise that this technique would give contrast on moiré samples, especially tBG which lacks the inversion asymmetry necessary to generate a piezo-electric response (17, 27, 28). Though PFM is expected to require closing an electrical loop between the AFM tip and the sample, published studies suggest that PFM in fact resolves the moiré contrast even on insulating substrates. In attempting to replicate the beautiful maps achieved by this technique, we stumbled upon torsional resonances, sensitive to dynamic friction at the AFM tip-sample interface, as being central to resolving moiré contrast.

## 1. Experimental

**Fig. 1.**
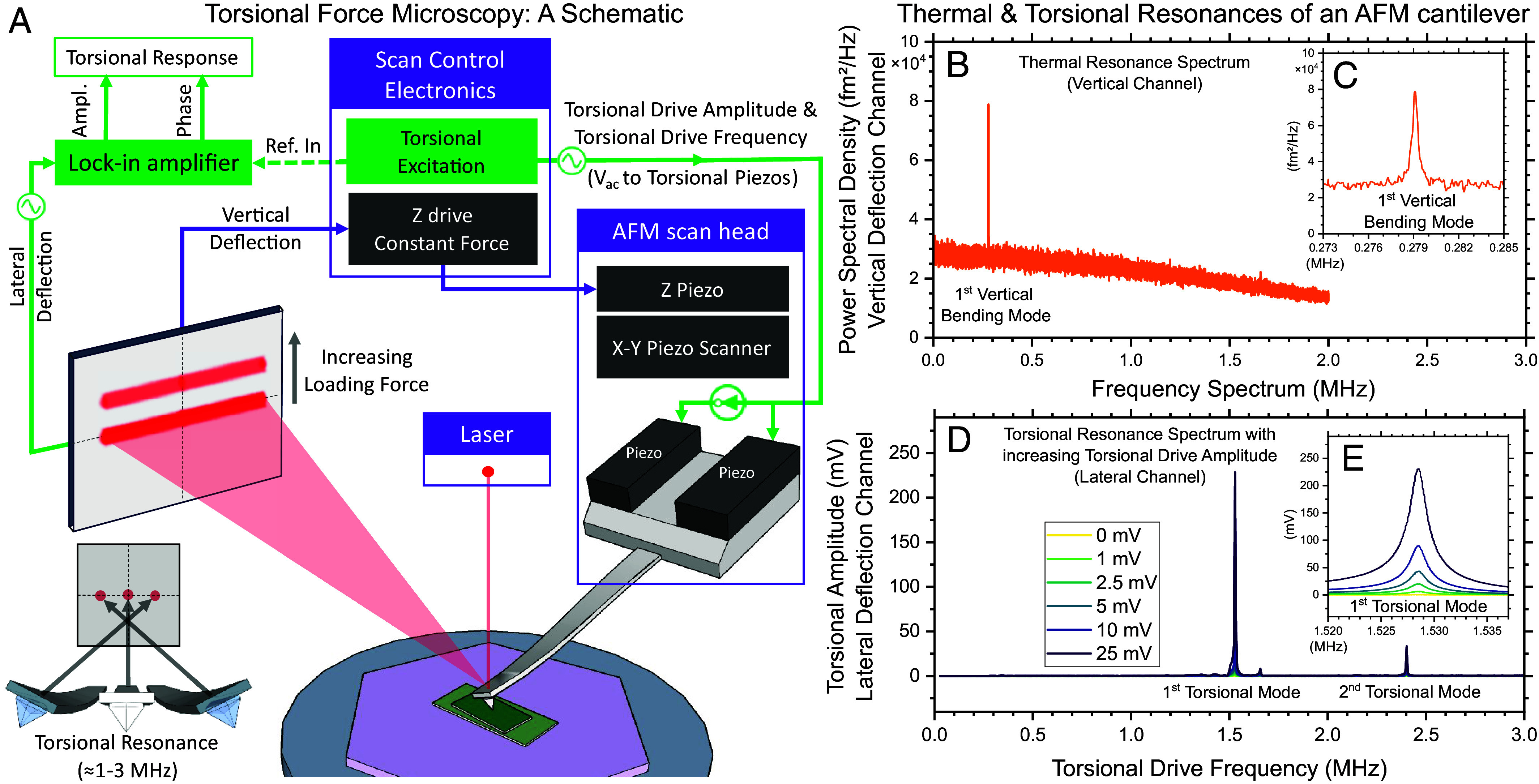
Torsional force microscopy (TFM). (*A*) Schematic diagram of TFM in a system with mechanically driven torsional resonances. An incident laser beam reflects off an AFM cantilever that is driven at a torsional resonance (typically 1 to 3 MHz). This torsional resonance is mechanically excited by applying an AC drive voltage to two piezos mounted in the AFM probe holder. We measure the amplitude and phase of the resulting lateral deflection signal on the photo-detector. A constant vertical loading force between the AFM tip and the imaging surface is maintained by a feedback loop that moves the AFM tip up and down according to the topography, as in contact-AFM. (*B*) Thermally excited resonance of an AFM cantilever (nominal spring constant 42 N/m) at room temperature in air, far from any surface, in ambient light, without any mechanical drive. The only measurable peak in power spectral density of the vertical deflection channel of the photo-detector is the fundamental resonance, i.e., the first vertical bending mode of the cantilever. (*C*) Zooming into a narrower frequency range shows that the resonance is at 279 kHz. (*D*) Torsional response of the same AFM cantilever (lateral photo-detector channel) as a function of drive frequency of the torsional piezos, for several drive amplitudes. Two prominent resonances appear at 1.529 MHz (*E*) and at 2.4 MHz, respectively.

In this work, we map spatial variations in torsional resonances of an AFM cantilever, in a technique we term torsional force microscopy (TFM). Fig. 1*A* presents a schematic diagram of the key components that enable TFM. The basic operation of TFM can be divided into two parts: First, a closed loop feedback (routed in purple arrows in Fig. 1*A*) tracks the topography to maintain a set vertical loading force; second, a torsional resonance is excited in the AFM cantilever and the mechanical response is measured in open loop (green arrows in Fig. 1*A*) The first closed feedback loop is identical to that used in contact AFM, LFM, or PFM, while the second open loop shares similarities with noncontact or tapping-mode AFM. The two loops operate in parallel. TFM does not require any electrical connections to either the tip or the sample, so the two can be electrically floating and insulating.

The bending of the cantilever as it moves into contact with the sample surface is measured as a change in the vertical position of the laser spot on a four-quadrant position-sensitive photodetector. Such a photodetector provides outputs proportional to the position of the laser spot along the vertical and horizontal axes. Thermal or mechanical drift and bimetallic expansion of coated AFM tips under the incident laser led to force offsets of the order of hundreds of nanonewtons over a few hours after aligning the laser on the cantilever. This drift, if not periodically checked and corrected, can damage both the AFM tip and the sample. We developed a protocol to accurately estimate the force applied by the AFM tip on the sample surface (*SI Appendix*).

In parallel to the closed feedback loop, an independent open loop maps spatial variation in the frictional response, revealing both moiré superlattices and atomic lattices. This open loop operates by mechanically exciting a torsional motion of the AFM cantilever, near a torsional resonance. Two piezos in the cantilever holder are driven 180^°^ out of phase with each other, to specifically excite torsional motion (Fig. 1*A*). This mechanical excitation of torsional resonance modes was pioneered by Huang and Su (29, 30). By sweeping torsional drive frequency, maxima in signal amplitude consistent with torsional modes of the AFM cantilever are measured (as an AC voltage) on the lateral deflection channel of the photodetector. The amplitude of this lateral signal in volts can be used to deduce the amplitude of torsional motion of the cantilever in nanometers, in turn enabling deduction of a lateral force—orthogonal to the vertical loading force (31).

Fig. 1*B* shows the thermal resonance spectrum (without any mechanical excitation) of an AFM cantilever measured in air at room temperature, far from any surface, on the vertical deflection channel of the photodetector. The *Inset* of Fig. 1*C* shows the first vertical bending mode of the AFM cantilever with a peak at 279 kHz. When a torsional drive is applied to the same cantilever, two resonances appear at 1.529 and 2.4 MHz in the lateral deflection channel of the photodetector, as shown in Fig. 1*D*. The amplitude of this response measured at the photodetector grows linearly with torsional piezo drive amplitude (Fig. 1*E*), reaching 225 mV at 25 mV torsional drive amplitude (each reported in units of zero-to-peak amplitude). For the range of cantilevers we tested, we typically see two such modes, the first between 1 and 1.6 MHz and the second between 1.4 and 3 MHz. Typically, the resonance with the highest ratio of response to drive was chosen for imaging. In the few instances when the second-most-prominent resonance was chosen, suitable results were still obtained. For a discussion on the nature of resonance modes being driven in TFM, *SI Appendix*.

By calibrating the lateral deflection sensitivity of the photodetector, the torsional resonance amplitude obtained in millivolts can be associated with side-to-side deflection of the tip apex in picometers (32–34). For a test AFM cantilever with a torsional resonance at 1.4 MHz, we extracted a deflection sensitivity of 3 pm/mV (or 3 nm/V), in line with values reported in the literature. Such sensitivities have substantial uncertainty (perhaps 30%), dominated by variation in height of tip relative to nominal values (32). For further details, including the estimation of peak-to-peak amplitude of torsional oscillation, *SI Appendix*.

A lock-in amplifier operating near the torsional drive frequency demodulates the measured torsional amplitude and phase at every pixel. Typical line scan speeds (each line consisting of both trace and retrace) ranged from 2 Hz over microns, to 4 Hz over hundreds of nanometers and up to 30 Hz over tens of nanometers. At these speeds, the lock-in amplifier input bandwidth was typically set between the lower end of 0.211 kHz (limited by electronics) to 10 kHz, with increasing bandwidth at increasing speeds, to avoid digitization. A standard operating procedure (SOP) to set up TFM is provided in *SI Appendix*.

## 2. Results

**Fig. 2.**
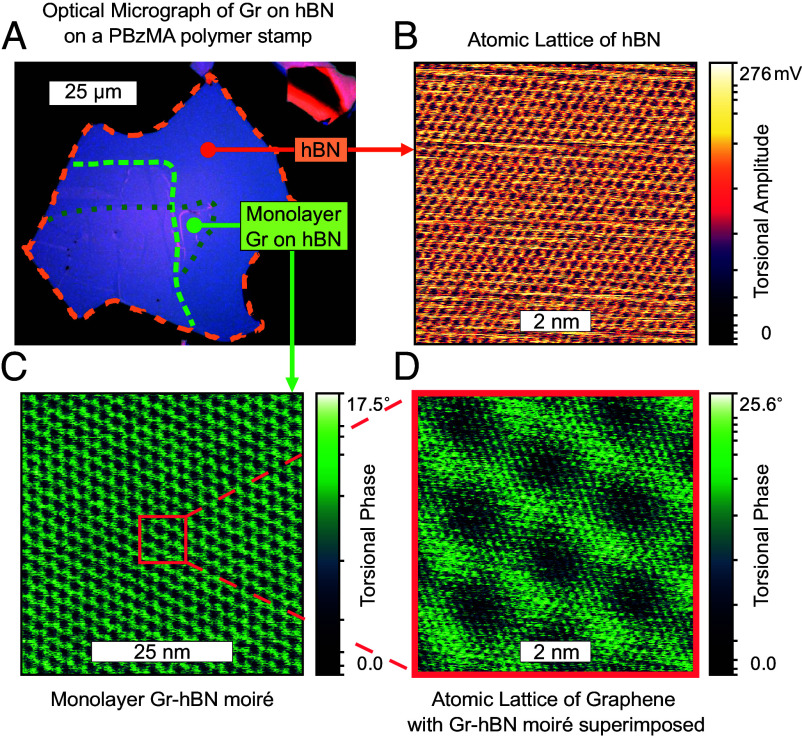
TFM of the atomic lattice of hBN & graphene & of a moiré of monolayer graphene on hBN (*A*) Optical microscope image of a graphene-hBN heterostructure on a multilayer polymer stamp with the color scale adjusted to highlight the contrast between graphene and hBN. The dashed outlines are a guide to the eye indicating the dimensions of the two graphene flakes (green) and the hBN flake (orange). (*B*) TFM image taken at the approximate location of hBN marked in (*A*) by an orange circle, shows torsional amplitude revealing the atomic lattice of hBN, at a force of 50 nN and a torsional drive of 2.5 mV at a speed of 24.4 Hz per line. (*C*) Moiré superlattice formed between monolayer graphene and hBN, measured at the approximate location marked in (*A*) by a green circle. A moiré period of 2.6 nm indicates a relative twist between monolayer graphene and hBN of 5.4^°^. (*D*) Higher resolution image taken from the center of (*C*). The fine granular features of the moiré in (*D*) are likely the underlying lattice of graphene. (*C* and *D*) were imaged at a force 100 nN and a torsional drive of 5 mV at a speed of 8.14 Hz per line. (*B*–*D*) were imaged with a 16× lateral signal amplifier enabled and at the 1.428-MHz torsional resonance, at a scan angle of 90^°^.

We now employ TFM to study a common VdW heterostructure of graphene on hBN. This sample was prepared in vacuum by picking up an exfoliated flake of hBN followed by graphene. Fig. 2*A* shows an optical microscope image of this open-face heterostructure. Fig. 2*B* shows the honeycomb atomic lattice of hBN as imaged by TFM. The atomic lattice could be measured with both the first and the second torsional resonances. Remarkably, commonly available AFM cantilevers (radius ≤ 25 nm) could be used without the need for sharp AFM tips, though sharp tips were preferred (radius ≤ 10 nm). To counter the effects of thermal drift, piezo creep, and piezo hysteresis, fast line scan speeds of between 10 and 30 Hz were used over square areas typically between 4 to 20 nm on a side. Extending the piezo scanning distance along the fast scan axis by 10% beyond the edge of the frame reduced the distortion in images. It is unclear whether the features correspond to true atomic resolution versus atomic lattice resolution (i.e., spatially averaged, eggcarton-on-eggcarton tip-sample interaction) (35). In any case, the ability to easily visualize the atomic crystal lattice in air at room temperature in a commercially available AFM has substantial implications for guiding stacking of atomically thin materials.

Fig. 2*C* shows TFM of a moiré superlattice formed between monolayer graphene and hBN with a period of a mere 2.6 nm. Here and throughout this manuscript, reported moiré periods are extracted from 2D FFTs. The clarity of the image highlights the impressive lateral resolution of TFM. Upon further zooming into the moiré structure, a periodicity consistent with the atomic lattice of either graphene or hBN emerged, superimposed on the moiré superlattice (Fig. 2*D*). *SI Appendix*, Fig. S2 shows the complementary TFM amplitude and phase images of Fig. 2. As the AFM tip is in direct contact with graphene while taking this image, the prominent atomic lattice is likely that of graphene. However, the vertical loading is sufficient that the underlying hBN lattice might be imaged. In addition to demonstrating the success of TFM in imaging the atomic crystal lattices of hBN and graphene, this result also confirms the sensitivity of TFM to moiré superlattices formed at the interface of monolayer graphene and hBN.

**Fig. 3.**
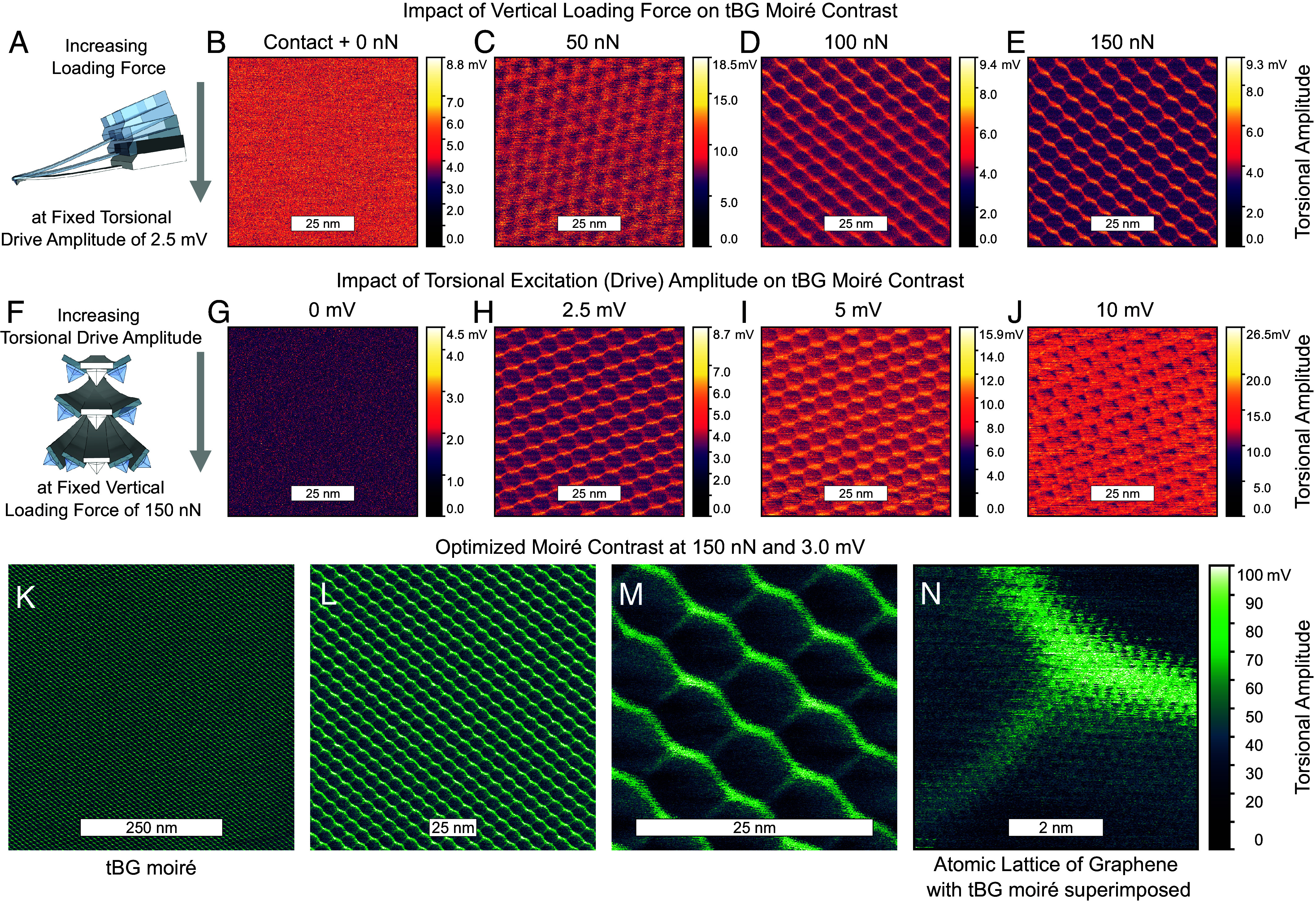
Imaging moirés in tBG: impact of vertical loading force & resonant torsional excitation amplitude on moiré contrast. (*A*) Schematic of increasing vertical loading force at a fixed torsional excitation. (*B*–*E*) TFM maps on the surface of tBG as force is increased from the minimum required to maintain contact of the AFM tip with the sample surface (Contact + 0 nN) to 150 nN in steps of 50 nN. A moiré superlattice is not visible at the lowest force but becomes stronger in contrast as force is increased. (*F*) Schematic of increasing torsional excitation amplitude at a fixed vertical loading force. (*G*–*J*) TFM maps with torsional drive amplitude increased from 0 to 10 mV. Though no moiré is observed at 0 mV, a moiré is clearly observed at 2.5 mV. Upon further increasing the drive amplitude, the moiré persists, but the contrast in torsional amplitude decreases, instead appearing as a change in torsional phase (*SI Appendix*, Fig. S3). (*K*) Using the near-optimal imaging parameters now determined for TFM, we image a larger region, revealing a moiré superlattice across 500 × 500 nm. The moiré period of 7.5 nm corresponds to a twist in tBG of 1.88^°^. (*L*–*N*) Subsequent images taken at higher resolution near the center of (*K*). (*M*) shows a fine granular detail accompanying the moiré which is revealed in (*N*) to resemble an atomic lattice, most likely of the uppermost graphene surface in contact with the AFM tip, with the tBG moiré superimposed. (*B*–*E* and *G*–*J*) were imaged at 1.4568 MHz with the lock-in amplifier bandwidth set to 102.6 kHz to ensure the resonance frequency was always within the input bandwidth. (*K*–*N*) were imaged at 1.4576 MHz, the peak of torsional resonance at 150 nN and at 3 mV drive amplitude, with the lock-in amplifier bandwidth reduced to 2 kHz and a 16× lateral signal amplifier enabled. (*B*–*E*, *G*–*J*, and *K*–*N*) were all imaged at 4.07 Hz line scan speed and a scan angle of 90^°^.

Next, we image a moiré superlattice formed in tBG. To establish reproducible conditions for imaging regardless of the AFM tip used, the sample being imaged, or other variables, Fig. 3 examines the impact of two key parameters of TFM: loading force and torsional drive amplitude. These two variables in turn control the tip-sample interaction. The tBG-on-hBN open-face heterostructure was prepared in vacuum, with an intended tBG twist angle of 2^°^. The period of the imaged moiré superlattice corresponds to a twist of 1.88^°^.

Moiré superlattices in tBG imaged using contact AFM techniques such as PFM and LFM have typically been reported for forces of 20 to 50 nN (17, 23). To study the impact of loading force, an accurate knowledge of the force applied is necessary (especially at low forces). We develop a protocol to accurately determine the force, starting by determining the minimum force required to keep the tip in contact with the sample. We refer to this baseline as “Contact + 0 nN.” Fig. 3*A*–*E*)shows a schematic and then TFM images acquired as increasing force from “Contact + 0 nN” to 150 nN in steps of 50 nN, at a constant torsional drive amplitude of 2.5 mV. Moiré contrast increases dramatically as force is increased. Next, the torsional excitation’s drive amplitude is increased, while keeping the drive frequency and force fixed. Though some torsional excitation is necessary, high contrast in measured signal amplitude is immediately apparent at very low drive amplitude. As drive amplitude is increased, the measured signal switches from amplitude to phase. The mechanism for this remains to be studied.

A force of 150 nN was not required on all tBG samples; moiré superlattices in tBG were successfully imaged at forces from 10 to 300 nN. Fresh AFM tips on fresh samples enabled mapping moiré superlattices at comparatively lower forces. With a sharp tip apex of a fresh tip, the pressure applied on the surface is likely much greater for a given force, so we speculate that the moiré contrast depends directly on the pressure applied, rather than the force. Samples likely accumulate a stubborn layer of adsorbates over months demanding higher forces for imaging through these layers. The tBG sample imaged in Fig. 3 was prepared over 5 mo prior to being imaged. It was mainly stored in a nitrogen drybox but was exposed to air for days at a time on multiple occasions.

As the force was increased from zero (not in contact with the sample) to the minimum required to remain in contact (“Contact + 0 nN”) and onward to hundreds of nanonewtons, the torsional resonant modes were observed to shift to higher frequencies. The measured amplitude of the resonance also reduced with increasing force, indicating damping of the resonance (30). The shift in frequency ranged from tens of Hz to tens of kHz depending on the force applied.

Once optimal parameters of force and drive amplitude were determined, the moiré superlattice in tBG was imaged at varying length scales. The lock-in amplifier bandwidth was also reduced to improve SNR. Fig. 3*K*–*N* show a tBG moiré with a period of 7.5 nm, imaged with successively reduced scan area, without changing any other settings. The smallest-scale map (N) covers portions of three moiré cells; superimposed on this moiré pattern is an atomic-scale periodic structure, likely the atomic lattice of graphene. These results point to the versatility of TFM in imaging both moiré superlattices and atomic lattices on the same sample, without having to change anything more than the frame size. *SI Appendix*, Fig. S3 shows TFM phase images corresponding to the amplitude images of Fig. 3.

Going forward, we continued to follow this protocol of first determining the minimum force required to remain in contact and then stepping up from “Contact + 0 nN” to higher forces until satisfactory moiré contrast is observed.

**Fig. 4.**
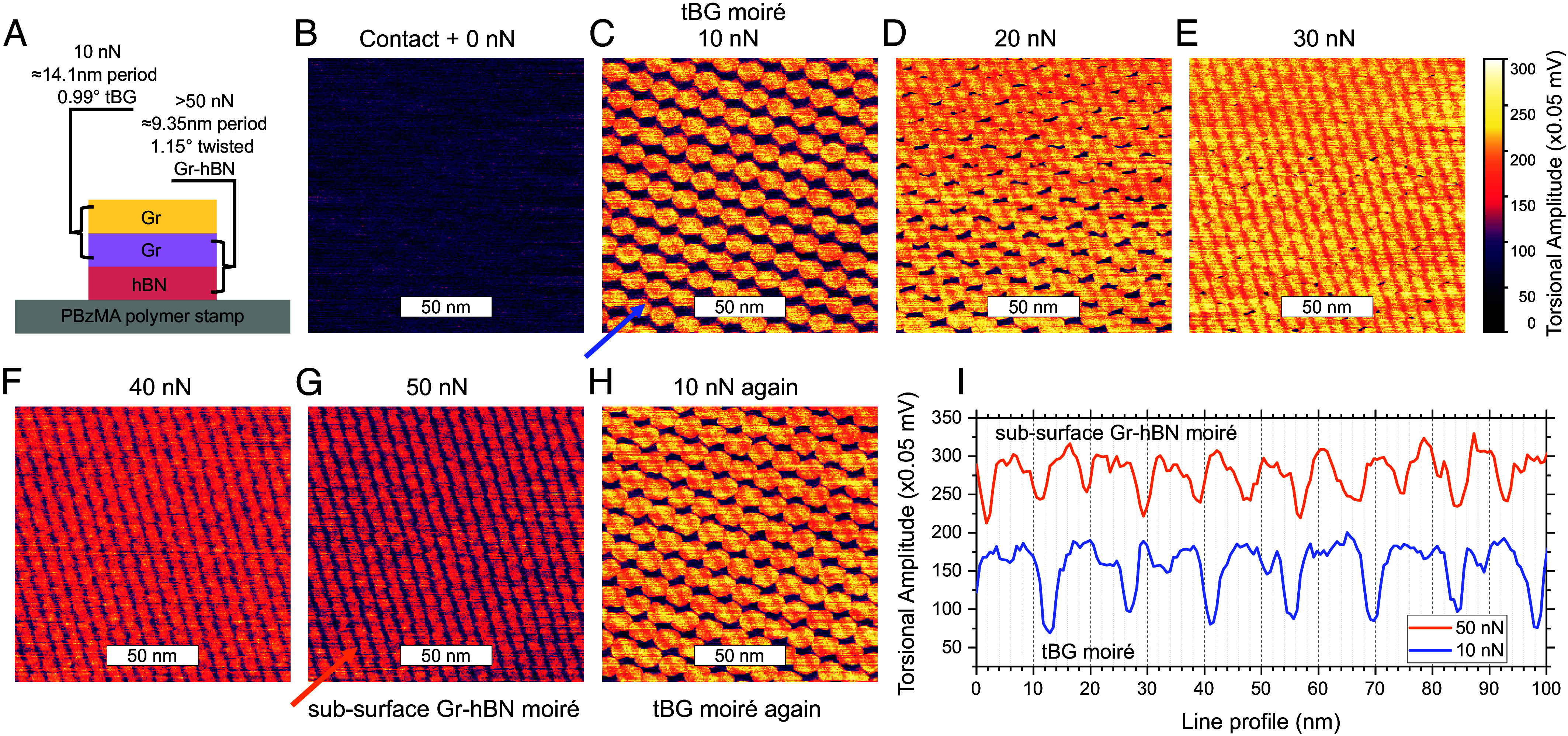
Imaging a subsurface moiré. (*A*) Schematic layer structure of the sample being imaged: tBG is stacked atop a flake of hBN on a multi-layer polymer stamp terminated with PBzMA. (*B*–*H*) are all imaged at the same location of the sample. (*B*) Resonant torsional amplitude at a vertical force equivalent to the minimum force required for the AFM tip to remain in contact with the surface (Contact + 0 nN). (*C*) Increasing the force to 10 nN reveals a well-defined moiré pattern with a period of 14.1 nm. (*D*–*F*) reveal a transition from the moiré observed in (*C*) to a moiré in (*G*) as force is stepped by 10 nN with each image. The moiré period observed in (*G*) at a force of 50 nN is 9.35 nm, different from the observed period in (*C*) at a force of 10 nN. (*H*) When force is reduced back down to 10 nN, the pattern returns to resembling that in (*C*), indicating the change in moiré from (*C*) to (*G*) is not a temporary surface cleaning effect. (*I*) Line profiles, taken along lines indicated by the blue (*C*) and orange (*G*) arrows. The low-force moiré is likely to be a tBG moiré. As force is increased, it is likely that the moiré formed by the subsurface graphene with the underlying hBN is revealed. The period of 14.1 nm of the tBG moiré corresponds to a twist angle of 0.99^°^. The period of 9.35 nm of the Gr-hBN moiré corresponds to a twist angle of 1.15^°^. These moiré periods are extracted from 2D FFT of the image and not their line profiles. All images in the series were acquired at the 1.3299-MHz resonance at 0.5-Hz line scan speed at a scan angle of 0^°^, with the 16× lateral signal amplifier enabled. The torsional piezos were driven indirectly by cross talk in the electronics by applying 500-mV drive amplitude—which would correspond to 10 to 12.5 mV of torsional drive amplitude applied directly to the piezos. The 10-nN and 50-nN line profiles are offset for clarity.

For one sample of tBG on hBN (schematic cross-section, Fig. 4*A*), the moiré superlattice observed at 10 nN (Fig. 4*B*) dramatically transformed as force was stepped up to 50 nN (*C*–*G*). Upon lowering the force back to 10 nN (*H*), the moiré returned to resemble the pattern observed at 10 nN prior to the force ramp. Line profiles (*I*) along arrows marked in (*C*) and (*G*) show two different periods for these images, suggesting that changing the applied force allows us to select which of two superimposed moirés to image.

The moiré seen at low applied force is likely that of tBG, whereas the moiré seen at high force is likely below the surface, presumably from unintentional rotational near-alignment of graphene on hBN. The period of the first moiré is 14.1 nm, corresponding to tBG twisted at 0.99^°^. This should be compared to the 1.3^°^ intended fabricated twist angle of the tBG. A twist relaxation of 0.3^°^ is often seen at these low twist angles (36–38). The subsurface moiré period of 9.35 nm corresponds to a graphene-hBN moiré at 1.15^°^ twist. These results indicate that increasing force nondestructively allows TFM to map a subsurface moiré. On many additional tBG samples, we have now seen a second moiré corresponding to an underlying hBN’s near-alignment to the subsurface layer of graphene.

This measurement was performed before we understood the mechanism for TFM imaging and the measurement was set up with excitation routed to the AFM tip, as is common in modes like PFM. We later found that due to cross talk, torsional piezos in the probe holder were driven with an excitation 2 to 2.5% of the amplitude applied to the AFM tip and that the AFM tip was disconnected from the electrical circuit. A detailed description of this issue and a comparison of the frequency spectrum in TFM mode (directly driven torsional piezos) vs PFM mode (cross talk–driven torsional piezos) is shown in *SI Appendix*, Fig. S1.

*SI Appendix*, Fig. S5 shows a 2 × 2 μm map of a tBG moiré with a spatially varying period of 44 to 51 nm, corresponding to twist angles around 0.3^°^, demonstrating that TFM can image nanometer-scale moirés over areas relevant to typical electronic devices. The moiré unit cells appear hexagonal, suggesting that the surface is unreconstructed despite the small twist angle (39, 40), though it is possible that TFM is not sensitive to the internal structure of the moiré unit cell. Though we mostly studied stacks made in vacuum, we also confirmed that TFM works on tBG-hBN samples prepared in air on PC stamps (*SI Appendix*, Fig. S6).

## 3. Discussion

We now examine the origin of both moiré and atomic lattice contrast in TFM. In LFM, a more commonly used technique, the AFM cantilever’s lateral deflection is measured on the photodetector as the tip is dragged along the surface. In TFM, the tip again rubs against the sample surface, now at a drive frequency near the MHz resonance of the cantilever, and changes in the resonant response are measured on the photodetector. By analogy, we suggest that the signal on the photodetector in TFM is a measure of tip-sample friction, as in LFM. This view is supported by our observation of increasing contrast with increasing vertical tip-sample force. Moiré contrast originating from friction has been reported to be velocity-dependent, so the higher tip-sample velocities in TFM may provide higher contrast for imaging 2D materials (41).

Though both the atomic lattice of VdW materials and their moirés have also been imaged with LFM (22), TFM offers several advantages. First, TFM adds the ability to image subsurface moiré superlattices. Second, like other finite-frequency techniques TFM is resilient to electronic noise outside of the lock-in amplifier’s input bandwidth. In comparison, LFM and contact AFM operate by summing the signal from DC to a few kHz (limited by a low pass filter) and are therefore strongly affected by 1/f noise. Third, TFM can work with a wide range of cantilevers, allowing applying high vertical forces where needed to enhance moiré visibility. LFM by contrast uses cantilevers with a very low spring constant (≪ 0.5 N/m), limiting the range of vertical forces that can be applied. Last, we have found that TFM can yield good contrast at any scan angle relative to the long axis of the cantilever (Figs. 2 and 3 used 90^°^ scan angle, and Fig. 4 used 0^°^) whereas LFM requires imaging at a fixed scan angle of 90^°^.

Next, we compare TFM with lateral PFM at contact resonance (or CR-PFM). Here as well, TFM offers several advantages. First, TFM can image a tBG moiré in a single two-dimensional scan. In contrast, CR-PFM has been reported to require superimposing two orthogonal images, with manual rotation of the sample in-between, to fully resolve the hexagonal unit cell of a large-period moiré superlattice. Second, TFM can image atomic lattices, which has not yet been reported for PFM. Last, TFM does not require a conducting AFM tip with bias applied between the tip and the sample, which PFM does require.

To implement TFM in instruments lacking the capability for mechanically exciting torsional resonances in an AFM cantilever, photothermal excitation of torsional resonances has recently been demonstrated to image the atomic lattice of graphite (HOPG) and to image structural features of living cancer cells, proving the versatility of the technique (42, 43).

TFM is closely related to torsional resonance microscopy (TRM), a technique described by Huang and Su nearly two decades ago (29, 44). But the distinctions are important. TRM feeds back on the torsional resonance amplitude and uses the deviation in this amplitude from its setpoint to move the Z-piezo, thus varying the vertical loading force. For imaging atomically thin materials placed on soft polymer stamps, TFM allows vertical loading force to be selected and kept steady, to balance moiré contrast (Fig. 3) against the risk of tearing the materials during imaging. Incorporating a phase-locked loop (PLL) to track torsional resonance frequency as it shifts due to tip-sample interaction has been demonstrated with TRM (45) and may offer advantages in TFM.

TFM, like other ambient-based scanning probe techniques, suffers from thermal drift, piezo creep, and piezo hysteresis in the lateral (X-Y) scan axis, complicating quantitative extraction of moiré period and thus twist angle and strain. Temperature- and humidity-controlled enclosures, as well as correcting for piezo creep and hysteresis (either actively during imaging or using sensor data post-imaging), should help reduce these errors. Additionally, with the difference between the lattice constants of hBN and graphene being within the calibration uncertainty for ambient scanning probe techniques, TFM alone cannot be used to identify which atomic lattice is being imaged—prior knowledge of the structure studied, or access to other probes, is necessary. Similarly, twist relaxation as well as unintentional deviation from the fabricated twist angle in tBG on hBN stacks makes attribution of the moiré observed to either of the two possible moiré systems (tBG or Gr-hBN), challenging, especially if only one moiré, of period less than 14.25 nm, has been observed.

## 4. Conclusion

An open secret in the field of VdW materials is the poor success rate of most scanning probe techniques at imaging moiré superlattices formed in tBG –a problem not shared with the moirés formed in Gr-hBN, which can be imaged rather easily in conventional tapping-mode AFM. Using the SOP developed for TFM, we were able to find at least one moiré in 94% of the 33 regions in 32 unique samples measured. Regions that did not show a moiré had likely relaxed to Bernal stacking. Atomic lattices were observed at an even higher success rate (see *SI Appendix*, *Note 5.C.* on duration and volume of study).

To summarize, we have demonstrated the use of TFM to image atomic crystal lattices and of moiré superlattices formed in VdW materials, in air at room temperature. Relying on dynamic friction at the tip-sample interface, with detection sensitivity enhanced by the torsional resonance of the AFM cantilever, TFM operates without the need for any electrical contacts to either the sample or the AFM tip. Thus, TFM can be applied to give tight feedback on the structure of synthesized VdW stacks, helping make such synthesis more controlled. Given the strong dependence of electronic band structure on the interlayer twist angle and its spatial variation, this could have a transformative impact on fundamental and applied research on VdW materials and devices. More broadly, TFM should find an application wherever imaging of frictional properties of surfaces gives insight into local structure. For example, TFM may enable imaging biological samples at extremely low forces, a niche hard to address with LFM.

## 5. Materials and Methods

### A. Sample Preparation.

Samples prepared in vacuum as part of this work used a robotic vacuum stacking tool based on one previously developed by one of the authors (46). Imaging was performed with the stack placed on a PBzMA-terminated multi-composition polymer stamp that was also used to pick up the hBN. This polymer stamp was prepared on a PDMS handle as described in the work just cited and was held on a clear quartz or sapphire substrate, during both stacking and imaging. Samples prepared in air as part of this work followed the technique developed by Sharpe et al., using a manually operated stacking tool (8). Imaging was performed with the stack still on a PC polymer stamp that was used to pick up the hBN and graphene. This polymer stamp was prepared on a PDMS handle and held on a clear glass slide, both during stacking and imaging. Post-fabrication, all samples were stored in a nitrogen drybox prior to being removed for AFM measurements.

### B. AFM Measurements.

All AFM measurements shown as part of this work were performed at Stanford University in a shared facility instrument at room temperature, in air, without any humidity or temperature control beyond the room’s air-handling, on a Bruker Dimension Icon AFM equipped with NanoScope V electronics and software version 9.40 (March 2019). As a confirmation, a few test measurements were also performed outside of the facility on a Bruker Dimension Icon AFM with NanoScope 6 electronics. No modifications were made to the hardware or the software of any of the instruments to perform these measurements. A DTRCH-AM probe holder (also used for PF-TUNA or TR-TUNA), with the tip-bias wire disconnected, was used to hold AFM tips. Various AFM tips were used to measure moiré superlattices and atomic lattices. Adama Innovations AD-2.8-AS & AD-2.8-SS, Oxford Instruments Asyelec.02-R2, and MikroMasch HQ:NSC18/Pt all showed good results. AFM images were analyzed in Gwyddion. A Standard Operating Procedure (SOP) for torsional force microscopy, to aid in the reproduction of these results, is provided in *SI Appendix*.

### C. Duration and Volume of Study.

A protocol that yielded the moiré superlattices imaged in this work was first developed in October 2022. Between November 2022 and June 2023, at least 33 regions on 32 unique samples were studied, of which 31 regions yielded at least one moiré (a success rate of 94%). Ten of these regions showed signs of two moirés, though a second moiré was not searched for and analyzed on all samples. Starting at the end of March 2023, torsional piezos were directly driven, as opposed to being indirectly driven by cross talk. Between April and June 2023, atomic lattices of hBN and graphene were searched for in at least eight regions of six unique samples, each of which yielded a discernible atomic lattice.

## Supplementary Material

Appendix 01 (PDF)

## Data Availability

All data, including raw AFM images, acquired from samples presented in this work are available at the Stanford Digital Repository (47).
