# Supplementary material for: Torsional force microscopy of van der Waals moirés and atomic lattices
Source: Proc Natl Acad Sci U S A. 2024 Mar 1;121(10):e2314083121. doi: 10.1073/pnas.2314083121 (PMC10927490; doi:10.1073/pnas.2314083121)
Supplement: Supplementary file 1 — Appendix 01 (PDF) [file pnas.2314083121.sapp.pdf]

Supporting Information for

Torsional Force Microscopy of Van der Waals Moirés and Atomic Lattices

Mihir Pendharkar, Steven J. Tran, Gregory Zaborski Jr., Joe Finney, Aaron L. Sharpe, Rupini V. Kamat, Sandesh S. Kalantre, Marisa Hocking, Nathan J. Bittner, Kenji Watanabe, Takashi Taniguchi, Bede Pittenger, Christina J. Newcomb, Marc A. Kastner, Andrew J. Mannix, David Goldhaber-Gordon

To whom correspondence should be addressed: Mihir Pendharkar, Marc A. Kastner, David Goldhaber-Gordon
E-mail: mihirpen [at] stanford.edu, mkastner [at] mit.edu, goldhaber-gordon [at] stanford.edu

This PDF file includes:

Supporting text
Figs. S1 to S10
Table S1
SI References

A comparison of TFM with other scanning probe microscopy modes

Table S1. compares some common scanning probe techniques used to image moiré superlattices in twisted bilayer graphene and to image atomic lattices in VdW materials, with TFM. The techniques compared are: Lateral Force Microscopy (LFM) and Friction Force Microscopy (FFM), Piezoresponse Force Microscopy (PFM), Conductive AFM (C-AFM), Amplitude-Modulated Kelvin Probe Force Microscopy (AM-KPFM), Frequency-Modulated KPFM (FM-KPFM), Scanning-Microwave Impedance Microscopy (S-MIM), Scanning Tunneling Microscopy (STM), AC mode AFM (AC-AFM), Force Modulation Microscopy (FMM) and Contact-Resonance AFM (CR-AFM), Torsional Resonance Microscopy (TRM) and Torsional Force Microscopy (TFM).

Table S1. Comparison of TFM with other scanning probe microscopy modes

SPM Mode	Topography Input (primary feedback loop)	Additional Input/Output	Measured Signal	Conductive Tip/Sample	Cantilever Driven[a]	tBG Moiré	Atomic Lattice
STM	Tunneling Current	-	Z-piezo motion	Yes	No	Yes	Yes
Contact AFM	Vertical Deflection	-	Z-piezo motion	No	No	-	Yes
LFM/FFM[b]	Vertical Deflection	-	Lateral Deflection	No	No	Yes	Yes
PFM[c]	Vertical Deflection	AC bias	Lateral Deflection (AC)[d]	Yes	No	Yes	-
C-AFM	Vertical Deflection	DC bias	Current through tip	Yes	No	-	Yes
S-MIM	Vertical Deflection	RF AC bias	Reflected RF signal	Yes	No	Yes	-
AC-AFM[e]	Vertical Deflection (AC)[d]	-	Z-piezo motion	No	Yes (Vertical)	-	Yes
AM-KPFM	Vertical Deflection (AC)	AC/DC bias	AC/DC bias	Yes	Yes (Vertical)	Yes	-
FM-KPFM	Vertical Deflection (AC)	AC/DC bias	AC/DC bias	Yes	Yes (Vertical)	Yes	-
FMM/CR-AFM	Vertical Deflection	Driven Vertical Resonance	Vertical Deflection (AC)	No	Yes (Vertical)	Yes	Yes
TRM	Torsional Deflection (AC)	-	Lateral Deflection (AC)	No	Yes (Torsional)	-	-
TFM	Vertical Deflection	Driven Torsional Resonance	Lateral Deflection (AC)	No	Yes (Torsional)	Yes	Yes

Notes:

a. In this table, a cantilever is described as driven when its motion is intentionally excited either mechanically using piezos or photothermally using laser light pulsed in a focused spot at the stem of the cantilever to cause local heating. A cantilever is not considered to be driven if the cantilever motion or fluctuations are induced by ambient room temperature (as in thermal resonance). Also, a cantilever is not considered to be driven if the motion originates only after the tip makes contact with the sample, even if such motion arises because of an applied AC bias between the tip and sample.

b. LFM requires a scan angle of 90°. Scan angle refers to the relative angle between the cantilever and the fast scan axis; an angle of 90° means the long axis of the cantilever is perpendicular to the fast scan axis. This enables LFM to sense local changes in the static surface friction as a change in the lateral bending of the cantilever, which is detected on the lateral deflection channel of the photodetector.

c. tBG moirés have been reported to be imaged by Lateral PFM (L-PFM). Hence, in this table the measured signal for PFM is noted as the AC component of lateral deflection. More broadly, PFM works with the AC component of both vertical and lateral deflection signals.

d. In this table, AC refers to the AC component of the signal. Where AC has not been specified, the DC component of the signal is used.

e. In this table, AC-AFM includes non-contact and intermittent contact AFM techniques. These include Non Contact-AFM (NC-AFM), Tapping mode AFM, Frequency Modulation-AFM (FM-AFM), Amplitude Modulation-AFM (AM-AFM), and Dynamic Force Microscopy (DFM), among others. These are in contrast with DC modes like Contact AFM and LFM where the AFM tip is in continuous contact with the sample surface during imaging.

Comments on the nature of the resonance modes being driven in TFM and their detection

For a simple cantilever, a horizontal rectangular beam supported at one end, torsional and lateral modes are generally expected at frequencies well above the first vertical bending mode. We might not expect to drive a purely lateral bending mode by torsional excitation, nor could we sense purely lateral cantilever motion on a photodetector, as the top surface deflecting the laser beam would not change its orientation. However, the rectangular beam of a typical AFM cantilever has a pyramidal tip projecting downward from near its end, so a nominally lateral bending mode would include a torsional component of motion, enabling such a mode to be both driven and detected (1–3).

In our analysis we treat the driven mode as primarily torsional. If it is primarily lateral, or has a significant lateral component, the tip motion could differ significantly from our estimate, since our calibration is based on calculating tip motion from torsional reorientation of the cantilever (4).

As a test, we imaged the atomic lattice of hBN with TFM and also performed a lateral deflection sensitivity calibration in the same experiment. The atomic lattice of hBN was first observed at nominally 5 nN of vertical loading force and a torsional piezo drive amplitude of 2.0 mV. At higher loading forces of 10 and 25nN, the image quality improved and the atomic lattice could also be imaged at higher torsional drive amplitudes. We now focus on the particular case of 5 nN and 2.0 mV. At these settings, the peak of the torsional resonance was measured to be at 1.456 MHz with a zero to peak amplitude of 100 mV, with a 16X signal gain amplifier enabled. The raw amplitude can hence be estimated to be 6.25 mV, prior to the 16X amplification. Since the thermal resonance was captured without the 16X amplification, 6.25 mV is the relevant point of comparison. For atomic lattice imaging, at these settings, a scan angle of 90 °was chosen along with a bandwidth of 10 kHz for the lock-in amplifier. A scan size of 12x12 nm with a scan speed of 12.2 Hz and 512 samples per line yielded acceptable results.

For this calibration, we used an Adama Innovations AD-2.8-SS AFM tip with a fundamental vertical (diving board) resonance at 50.6 kHz as measured by "thermal tune". The cantilever width and length were measured with an optical microscope, and the thickness and height of tip apex were approximated using the nominal values provided by the manufacturer. Additionally, the thermal resonance spectra were acquired for both the vertical and lateral deflection channels using the "high speed data capture" function for use in the analysis. The data were analyzed in a Jupyter notebook.

From this test we find the lateral deflection sensitivity to be 3 pm/mV and using the peak torsional deflection amplitude of 6.25 mV, we approximate the peak to peak spatial deflection of the tip apex to be 40 pm, during imaging with the above specified parameters.

A step by step procedure to perform this calibration has been described at the end of the SOP. Additional information and raw data have also been made available (5). These include: (1) the relevant software settings and workspace for acquisition of thermal resonance spectra of the lateral and vertical deflection channels (.bag and .wks), (2) thermal resonance spectrum acquired using the high speed data capture function (with vertical deflection as Channel A and lateral deflection as Channel B) (.hsdc), (3) a "thermal tune" spectrum of the fundamental vertical resonance (.txt), (4) TFM image of the atomic lattice of hBN at 5 nN and 2.0 mV drive amplitude (.spm), (5) a spectrum of driven torsional resonance at 1.4 MHz, as was used for imaging (.dat) and, (6) the Jupyter notebook used to analyze the data (.ipynb and .pdf).

AFM measurements:
A comparison of Torsional Resonance in TFM vs Piezoresponse Force Microscopy (PFM) Modes

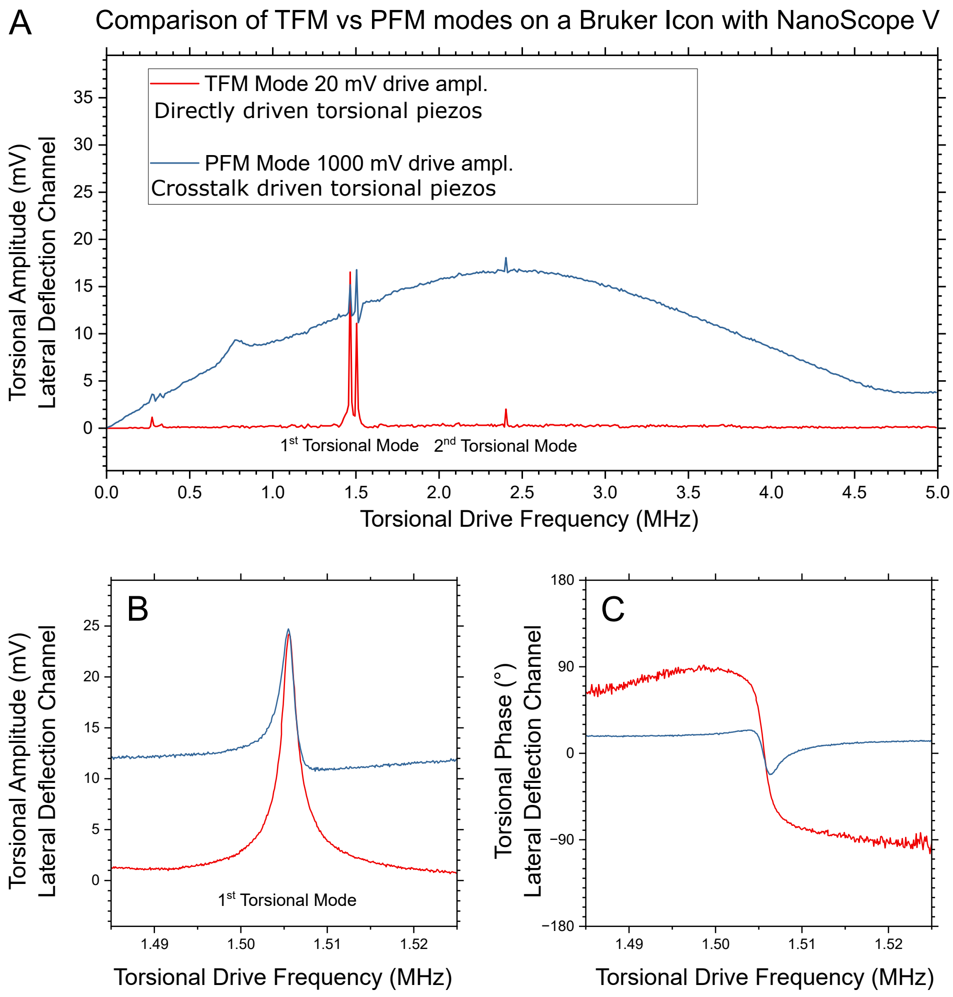

Fig. S1. Comparison of TFM vs. PFM mode of driving the torsional piezos The torsional resonance spectrum from near 0 to 5 MHz is shown in the blue curve in (A) and was measured in the PFM mode by parasitically driving the torsional piezos and selecting a "tip bias" of 1 V. The curve shown in red in (A) shows this resonance spectrum in the TFM mode, acquired by applying 20 mV directly to the torsional piezos (applying the excitation in PFM mode required 50x higher voltage, 1V at 1.5 MHz.) (B) and (C) show a zoom-in of the first torsional mode in amplitude and phase respectively. These comparison measurements were performed in air, without any sample in proximity of the AFM tip, on an ASYELEC.02-R2 AFM tip with a nominal first vertical bending mode at 285 kHz and a nominal spring constant of 42 N/m. For the particular AFM tip chosen here, there is a split resonance at the first torsional mode, possibly due to the properties of this particular AFM cantilever or its mechanical clamping in the probe holder. (B) and (C) zoom into one peak of that split resonance.

Prior to March 2023, our TFM measurements were performed by selecting piezoresponse force microscopy (PFM) mode in the Bruker Dimension Icon NanoScope software. However, because of the way we configured hardware electrical connections *no bias was actually applied to the tip.* Instead, through crosstalk in the electronics, torsional piezos on the probe holder were driven with amplitude of 2-2.5% of the tip bias we selected (20-25 mV of 1V). When the drive frequency was close to a MHz torsional resonance, this drive amplitude mechanically excited torsional motion in the AFM cantilever. Details: The standard DTRCH-AM probe holder we used has two special features compared to most probe holders: torsional piezos and an electrically-insulating Macor AFM probe seat. In the software, we chose typical PFM settings: routing an AC bias voltage to the AFM tip, and electrically grounding the sample. However, the normal outcomes of these settings were defeated in hardware, as explained below. There are 4 electrical traces on this probe holder's PC board. A photo of the probe holder is shown in Fig.S7(A), though these traces are not visible. Three of the traces connect to the torsional piezos to drive the two piezos 180° out of phase with each other, and the fourth trace comes to a dead end on the PCB. Software configures which signal is routed through the scan head to each trace. The prominently-visible white wire bypasses the insulating probe seat to connect electrically to the tip without routing through the scan head. We verified that disconnecting this white wire and taping it to the side of the instrument (Fig.S7(B)) had no effect on image contrast or the observed torsional resonance. Nor was the sample effectively grounded, as it was mounted on a polymer stamp atop a 0.5 mm thick quartz or sapphire substrate. The moiré image contrast we nonetheless observed when our AC drive frequency matched a torsional resonance of the cantilever was thus not from a piezoelectric response of the sample. We nonetheless refer to this as a PFM configuration because of the

mode selected in software.

In PFM mode, the software-selected tip bias is routed on this probe holder to the trace that dead-ends on the PCB, while the white wire is not driven. Within the AFM scan head and the NanoScope V electronics, the wire that connects to this dead-end tip bias trace likely runs very close to the torsional piezo drive lines. Electrical crosstalk was found to carry over about 2-2.5% of the applied AC bias from the tip bias wire to the torsional piezos (2% at 1.5 MHz as shown in Fig. S1(B)), so telling the system to drive the tip electrically did not in fact bias the tip, but did parasitically drive the torsional piezos.

In a newer version of the electronics (NanoScope 6), this crosstalk was found to drop to about 1% based on a direct measurement of the voltage on the wires leading to the torsional piezos in the DTRCH-AM probe holder.

Drawbacks of performing TFM measurements in PFM mode include: 1. Crosstalk is not an intentional part of the instrument design, so its amount may vary between instruments and probe holders (even with the same model number.) 2. The crosstalk-driven resonance in PFM mode produced an exceedingly high background signal as measured in the lateral deflection channel, leading to a poor SNR (see Fig. S1(A)&(B)). 3. The PFM mode lacks some very useful software tools that are provided in the TR mode, which is intended for operating torsional piezos: a. option to balance the left and right piezos to optimize driving torsional resonances of a cantilever. b. option to check whether the torsional signal observed is due to torsional motion or due to unintentional coupling of vertical motion into the lateral deflection channel of the photodetector. For these reasons we have transitioned to enacting TFM explicitly in TR mode, instead of nominally performing PFM and relying on crosstalk to excite a torsional resonance.

AFM measurements: Additional results

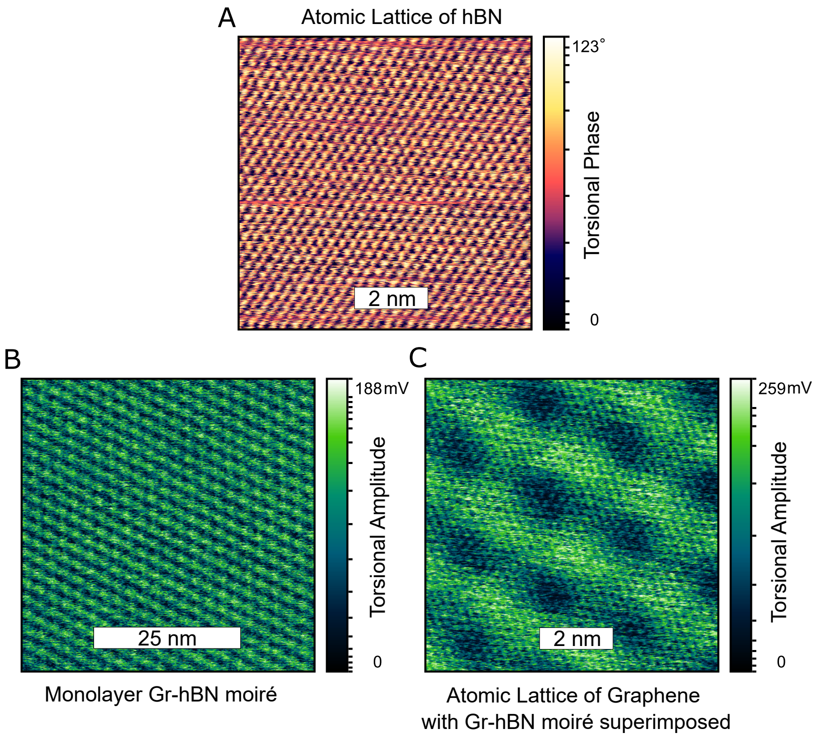

Fig. S2. Atomic Lattice of hBN & Gr and moiré of monolayer Gr-hBN Continuing from Fig. 2 of the main text, (A) shows the torsional phase corresponding to the torsional amplitude shown in Fig. 2 (A). (B) and (C) show the torsional amplitude of the torsional phase images shown in Fig. 2 (B) and (C). Images shown here and in Fig. 2 were processed using the align rows function in Gwyddion using polynomial fitting, followed by fixing zero to the bottom of the scale. The data was then plotted on an adaptive color scale to enhance lattice contrast. Imaging was performed using an Adama Innovations AD-2.8-SS AFM tip with a ratio of response to drive of 15 mV/mV when measured in air and away from the sample, at the 1.42 MHz resonance.

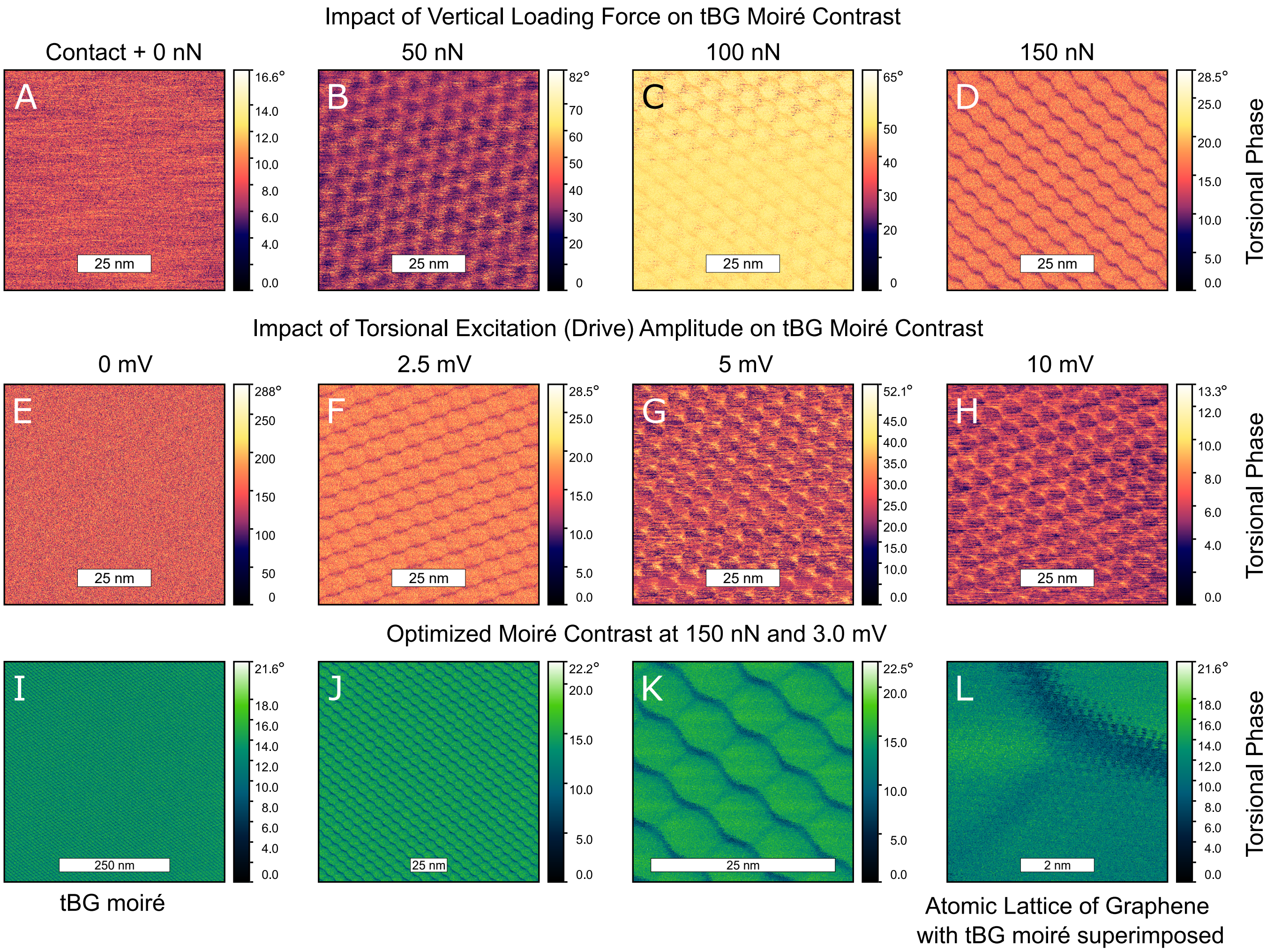

Fig. S3. Imaging moirés in tBG: Impact of Vertical Loading Force & Resonant Torsional Excitation Amplitude on Moiré Contrast Continuing from Fig. 3 of the main text, (A-L) are torsional phase counterparts of the torsional amplitude images shown in Fig. 3 of the main text. Images shown here and in Fig. 3 were processed using the align rows function in Gwyddion using polynomial fitting, followed by fixing zero to the bottom of the scale. The data were then plotted on a linear color scale. Data scale for main text Fig. 3 panels (K-N) has been restricted uniformly to 100 mV to provide a direct perspective of how the image evolves as zoomed in at the same imaging settings. Imaging was performed using an Adama Innovations AD-2.8-SS AFM tip with a ratio of photodetector response to piezo drive of 30 mV/mV when measured in air and away from the sample, at the 1.45 MHz resonance. At the time of imaging, the sample was over 5 months old and no prior surface cleaning or treatment was performed on this sample. Typically, the sample was stored in nitrogen environment with frequent removal to air for days during AFM imaging sessions.

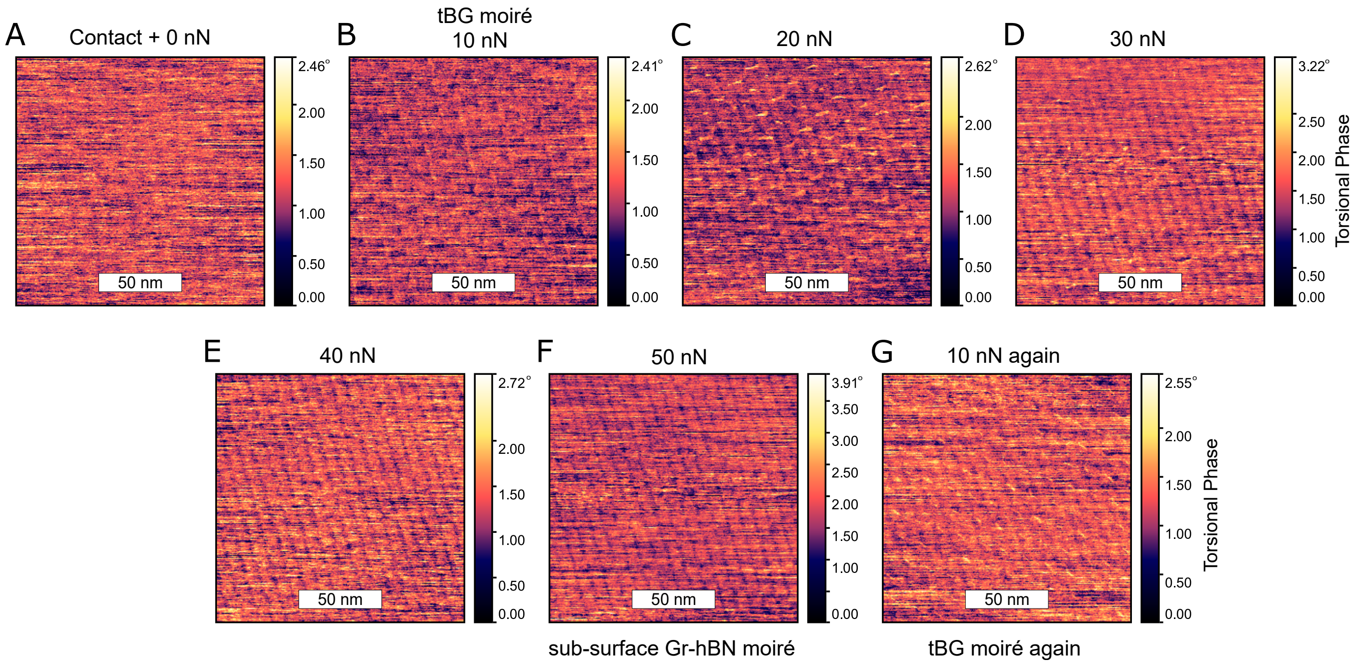

Fig. S4. Imaging a subsurface moiré Continuing from Fig. 4 of the main text, (A-G) are torsional phase counterparts of the torsional amplitude images shown in Fig. 4 of the main text. Images shown here and in Fig. 4 were processed using the align rows function in Gwyddion using polynomial fitting, followed by fixing zero to the bottom of the scale. The data were then plotted on a linear color scale. Data scale for main text Fig. 4 panels (B-H) has been restricted uniformly to 300x0.05 mV to provide a direct perspective of how the image evolves as force is stepped at the same imaging settings. Imaging was performed using an Adama Innovations AD-2.8-AS AFM tip with a ratio of response to drive of 0.125 mV/mV when measured in air and away from the sample, at the 1.32 MHz resonance. The lower ratio of response to drive is due to the image being taken with indirectly- rather than directly-driven torsional piezos.

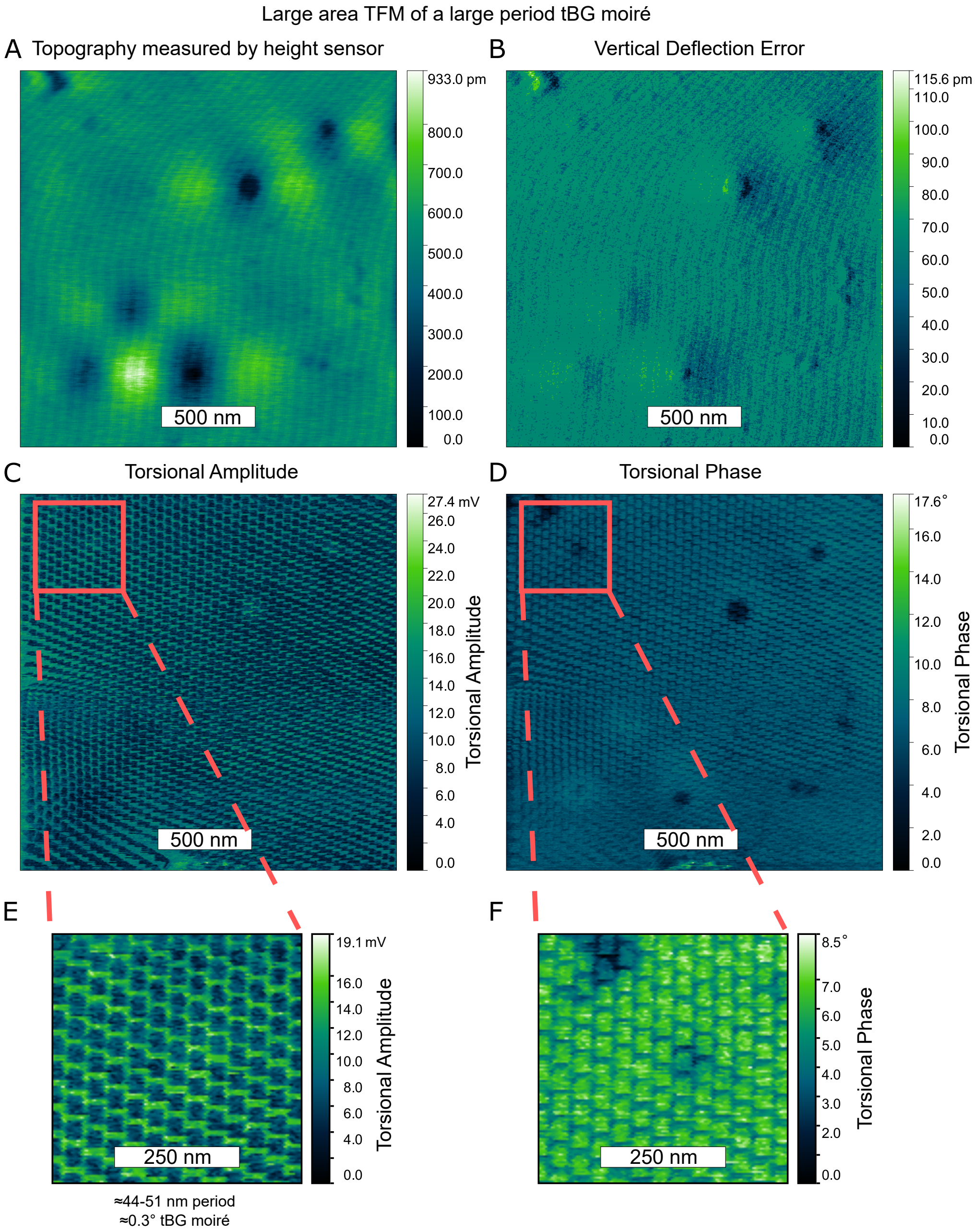

Fig. S5. Large area TFM of a large period tBG moiré superlattice TFM was tested on a tBG stack (prepared in vacuum) over a 2x2 μ region –dimensions relevant to electronic devices. (A) shows the topography measured by a height sensor and (B) shows the vertical deflection error as compared to the deflection setpoint. The faint parallel lines in (A) and (B) were determined to be periodic instrument noise. (C) and (D) show the TFM amplitude and phase, respectively. (A-D) were acquired simultaneously as separate measurement channels of the same scan. The variation of the moiré period over the area imaged is apparent in (C) and (D). (E) and (F) are digitally zoomed-in views of (C) and (D), respectively. The moiré period in (E) varies from about 44-51 nm, corresponding to a twist angle of 0.3° in tBG. Images shown here were processed using the align rows function in Gwyddion using polynomial fitting, followed by fixing zero to the bottom of the scale. The data were then plotted on a linear color scale. The scan parameters were: Loading force of nominally 200 nN with a torsional drive amplitude of 50 mV at the 1.210 MHz torsional resonance and a line scan speed of 1.0 Hz. The scan angle was 90° and a 16x signal amplifier was enabled. Imaging was performed using an Oxford Instruments ASYELEC.02-R2 AFM tip with a ratio of response to drive of 10 mV/mV when measured in air and away from the sample.

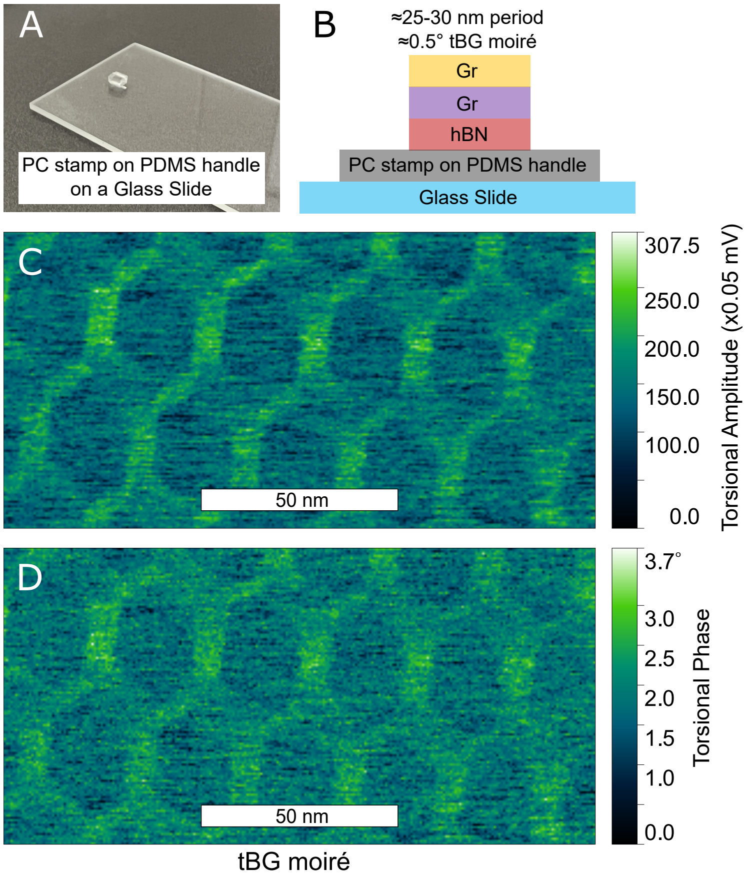

Fig. S6. TFM of tBG on a PC stamp placed on a PDMS handle on a glass slide To confirm the operation of TFM on VdW heterostructures held with other common stamp surfaces, a tBG sample prepared in air on a Poly(Bisphenol A carbonate) (PC) stamp was imaged. During both stacking and imaging, this sample was held on a PDMS handle placed on a glass slide. These results show the versatility of TFM and its use in rapid feedback on stack synthesis: PC-on-PDMS-stamps held on a glass slide are commonly used to stack multiple successive VdW layers. The moiré period is about 25-30 nm with a computed corresponding twist angle of 0.5°. This measurement was performed with indirectly driven torsional piezos. Images shown here were processed using the align rows function in Gwyddion using polynomial fitting, followed by fixing zero to the bottom of the scale. The data were then plotted on a linear color scale. The scan parameters were: Loading force of nominally 50 nN with a 1000 mV drive amplitude at the 1.294 MHz torsional resonance and a line scan speed of 0.5 Hz. The scan angle was 0° and a 16x signal amplifier was enabled. Imaging was performed using an Adama Innovations AD-2.8-AS AFM tip with a ratio of response to drive of 0.011 mV/mV when measured in air and away from the sample. For this particular sample, the moiré contrast improved as force was stepped up from "Contact + 0 nN" to 75 nN, at which point the high force led to a tear in the sample. The tear likely occurred due to a sharp AFM tip and a comparatively soft polymer structure (PC-on-PDMS as compared to our vacuum-compatible lithographically-patterned stamps.) When using a new type of stamp or AFM tip, force must be stepped up carefully and the effect on images examined. Once acceptable moiré contrast has been obtained, any further increase in force to enhance contrast must be approached with caution.

Torsional Force Microscopy: a Standard Operating Procedure (SOP) to image VdW moiré superlattices and atomic lattices

This SOP aims to provide the necessary guidance to enable the reader to replicate the results of this work performed on a Bruker Dimension Icon AFM with NanoScope V electronics at Stanford University. It has been written assuming the reader has a basic working knowledge of operating the instrument in contact and non-contact AFM modes. This SOP is merely a suggested set of steps and not a substitute for instrument manuals, or for taking care to secure the safety of the instrument, samples, and/or users. As this protocol evolves, an updated version of this SOP may be made available (5). Additional information about setting up the instrument for torsional resonance can be found elsewhere (3).

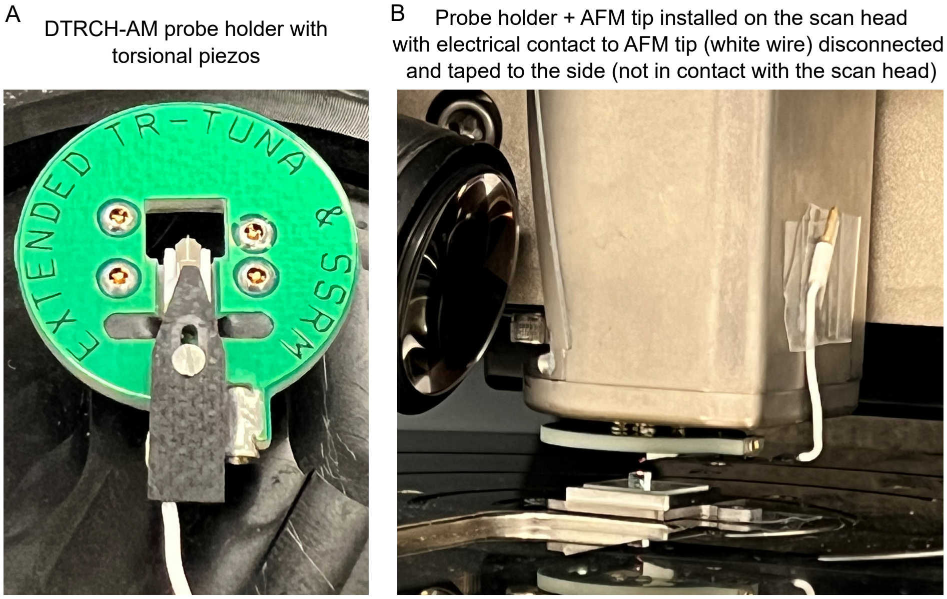

Fig. S7. Torsional probe holder (A) DTRCH-AM torsional probe holder with an AFM tip inserted. The tip is seated on an electrically insulated piece of Macor, under which the two torsional piezos are housed. A clamp (in black) holds down the AFM tip and is also the only electrical contact to the AFM tip with a white wire connected to it. (B) Probe holder installed on the scan head, with the white wire taped to the side of the scan head to ensure it doesn't make contact with the chassis.

A. Getting Started.

A.1. An "ingredient list" for TFM:.

1. Probe holder with torsional piezos.

   (a) An AFM probe holder with torsional piezos, to mechanically excite the torsional resonance, is required for TFM. We worked with a DTRCH-AM probe holder (see Fig.S7(A)).

   (b) Any signal preamplification box or other associated hardware is not required for TFM and does not need to be installed.

2. AFM tips.

   (a) Adama Innovations AD-2.8-AS & AD-2.8-SS, Oxford Instruments Asyelec.02-R2 and MikroMasch HQ:NSC18/Pt have been tested to work, including for atomic lattice imaging.

   (b) AFM tips resistant to wear were preferred since some experiments required application of nominal forces exceeding 100 nN.

3. Sample to be imaged.

   (a) For tBG and Gr-hBN samples, a fresh sample and a fresh AFM tip were not found to be necessary for imaging a moiré superlattice, but moiré contrast was often visibly improved with both a fresh sample and a fresh tip.

   (b) For atomic lattice measurements of hBN, samples as old as a few years and stored in air showed a discernible atomic lattice. Atomic lattices of other materials may behave differently. Fresh samples are preferred for determination of initial imaging settings.

A.2. Setting up the hardware for TFM:.

1. Put tip in probe holder & install the probe holder.

   (a) The DTRCH-AM probe holder places the AFM cantilever on a Macor seat that electrically isolates the cantilever from the rest of the probe holder. The cantilever is secured by a clamp which has a white wire that provides the only electrical contact to the AFM tip (see Fig.S7(A)). This white wire is left disconnected and taped to the side of the scan head such that the tape ensures the metal end of the wire does not come in contact with the chassis of the AFM as shown in Fig.S7(B).

   (b) It may be best to center the AFM tip in the white Macor seat of the probe holder.

   (c) The screw on the clamp, holding down the AFM tip, should be reasonably tight. Overtightening dampens the resonance. It may take a few tries with the same AFM tip and comparing torsional resonances, to fully gauge the optimal setting.

   (d) We also aimed to put the AFM tip in the center of the field of view of the camera when it was fully zoomed out (no digital zoom) and aimed to align the long axis of the cantilever parallel to the camera image frame. The incident laser was found to be most focused near the center of the field of view with significant distortions at extremities. Results may vary dramatically if the laser is not focused on the AFM tip.

   (e) An anti-static gun for the tip and the sample may be useful but was not tested.

   (f) While no drawbacks are anticipated spurious voltages on scan head may affect the electrostatic potential at the tip sample interface and worse, may change during scan affecting imaging conditions.

2. Put the sample in place & turn on the vacuum.

   Note: Double-sided tape to hold the sample was not tested and may not be well suited.

A.3. Setting the NanoScope software for TFM.

1. Creating a Logbook.

   (a) A table with the following columns, created in an online or offline spreadsheet, accessible post-imaging, should suffice.

   (b) This log will be called upon in the following steps as "Make a logbook entry" without further details. All applicable fields should be entered at that time and will be required for accurate estimation of force during imaging, though it would be best to enter all possible values as often as one can.

   (c) The log should have the following eight columns:

      i. Time

      ii. Signal Sum (volts) – only an approximate value will be available after imaging begins.

      iii. Vertical Deflection (volts) – here, this represents the free vertical deflection of the cantilever in air, far from the sample. The value displayed in the software after imaging begins will not represent this free deflection and hence the vertical deflection column should be left blank after imaging begins. The feedback loop will try to ensure the vertical deflection is the same as the setpoint.

      iv. Horizontal Deflection (volts) – here, this represents the free horizontal deflection. Since there is no feedback loop that relies on this value, a drift of this deflection should not have immediate consequences to image quality. Yet, for developing an understanding of instrument drift, logging this deflection is necessary.

      v. Flying Condition (volts) – this represents the deflection setpoint, in volts, at which the tip retracts from the surface when imaging, i.e. "flies away". This will be a proxy for vertical deflection during imaging albeit it may be affected by electrostatics due to the proximity of the sample surface. The tip can be considered withdrawn when the "z-piezo" indicator in the software turns red and shows the tip has moved all the way up. Recording this flying condition would require pausing imaging and reducing the voltage setpoint every 30-60 minutes to determine how much the free vertical deflection has truly drifted by.

      vi. Last Point of Contact (volts) – this represents the deflection setpoint at which the tip is still in contact but any further reduction of setpoint makes the tip retract (fly away) from the surface. This will be logged in conjunction with the flying condition above. This deflection setpoint will also be referred to as "Contact + 0 nN" as it is the minimum force required to remain in contact with the sample in addition to any force that may be required to remain in contact.

      vii. Snapback (volts) – this represents the deflection setpoint at which the tip returns to making stable contact with the surface (the piezo indicator should be roughly in the middle of its range and green) after having previously retracted from the surface. Due to attractive/repulsive interactions between the tip and the sample, this deflection setpoint will differ from the last point of contact, but not by more 10s of nN.

      viii. Notes – enter comments like "Laser aligned", "enclosure closed", "about to click engage", "approached on hBN", "on tBG", "retracted", etc.

2. Starting "Torsional Resonance" experiment.

   (a) This experiment will now configure the electronics to directly drive the torsional piezos.

   (b) When setting up for the first time, select "Tapping Mode" followed by "Tapping Mode in Air", then "Torsional Resonance (TR-Mode)" and click "Continue".

   Note: Due to the number of settings that have to be changed, it is best to save the experiment once it has been correctly configured and then re-open the saved experiment every time afterwards. There is a step below to save the experiment for opening in future runs.

   (c) When opening a saved experiment:

      i. Select Cancel on the experiment selection window that pops up.

      ii. From the "Experiment" menu in the row up top, select "Open experiment" and open the saved TFM experiment.

      iii. The steps below on configuring the experiment in "Scan window" and configuring the "Engage settings" can now be skipped as they will be recalled with the saved experiment. Jump to "Align the laser on the cantilever".

3. Scan window.

   (a) With the experiment now open, it will begin with the "Scan" option selected from the column on the left where "Scan", "Engage" and "Withdraw" are show. If not, select "Scan" from that column.

   (b) Set the file name for scans and select the user data folder.

   (c) In the list of scan settings, right click on white space and select "Show all" to show all the previously hidden scan settings.

      i. With all scan settings visible, go to "Other", "Microscope Mode" and select "Dynamic Friction". This configures the instrument to operate in a contact AFM like mode with a feedback loop maintaining a constant vertical deflection setpoint, irrespective of the torsional resonance settings.

      ii. Next, go to "Scan", "XY Closed Loop" and set it to "Off". This will make all motions of the piezos inaccurate but is necessary for fast line scan speeds. Instead, the X-Y sensor data can be recorded as separate channels to correct for the inaccuracy, as much as possible, in postprocessing.

      iii. Ensure that under "Torsion", "TR Mode" is be set to "Enabled".

      iv. Ensure full Z range of the fine piezos is shown in "Limits", for "Z limit" and "Z range". This should be about 13-14 μm. If not, entering 15 (out of range) should automatically ensure these values are set to their maximum.

      v. Ensure "Amplitude Range" and "TR Amplitude Range" are both "4000 mV".

   (d) Next, the "Generic Sweep" button should be visible in the left most column. If it is not visible, from the options on the top of the window, select "Experiment" and configure the experiment environment to make "Generic Sweep" appear. Generic sweep will be used extensively during imaging to track the torsional resonance.

4. Engage Settings.

   (a) From the top of the window, select "Microscope" and select "Engage Settings".

   (b) In the "Engage Parameters" window that pops up, right click in the white space and select "Show All".

   (c) In "Stage Engage" options, for "SPM Engage Step", type in 0.02 μm. This will automatically set the minimum approach step to a value of about 0.035 μm (35 nm per step; the value of 20 nm previously entered was out of range and hence the minimum value was automatically selected). This can be increased to 100 nm if the approach is too slow.

   (d) With "Sample clearance" being set to 1000 μm and "SPM safety" set to 100 μm, it takes about 1-2 minutes to make contact with the surface with 35 nm per step.

   (e) Ensure in "Smart Engage" the "Engage Mode" is set to "Standard".

   (f) Do not change any other parameters. Incorrect parameters or a step size of microns in TR mode can lead to sharp tips damaging the surface of soft polymer stamps and also becoming blunt in the process.

5. Align the laser on the cantilever

   (a) Select "Setup", in the left column, to manually align the laser on the cantilever.

   Note: Since the amplitude of torsional motion is the highest near the free end of the cantilever (on the same end as the AFM tip), the laser should be positioned as close to the free end, while still ensuring a high value of signal sum. Values typically in excess of 5 volts were common for Au coated diamond probes and in excess of 4 volts for Ti/Ir coated probes.

   Note: A focused laser spot may be 40×25 μm with additional lower intensity spots on either side of this ellipse.

(b) Select the correct AFM tip from the list of AFM tips.

   i. Note the deflection sensitivity in nm/V and the nominal spring constant of the AFM tip in N/m or nN/nm

   ii. Thermal tune or other techniques can be used to estimate the above two parameters more accurately, if needed, but were not available in our version of the software within the torsional resonance mode.

   iii. Estimate the deflection in mV, per nN of force applied. It is the inverse of the value obtained by multiplying the deflection sensitivity and spring constant (nm/V $\times$ nN/nm = nN/V). For example, for a deflection sensitivity of 60 nm/V and a spring constant of 2.8 nN/nm, this comes out to about 6 mV/nN. This means that if the force has to be increased by 100 nN, the deflection setpoint must be increased (made more positive) by 600 mV.

(c) Move to the alignment station.

(d) Align the laser on the AFM tip to 0 volts (0,0) on both vertical and horizontal deflection indicators.

(e) Make a logbook entry.

Note: Do not move the laser or mirror deflection knobs once the laser is set as close to 0,0 as possible. The laser will heat up the cantilever and the vertical deflection value will start to drift (almost immediately) towards a more negative or positive value. This drift is expected and should reach a steady state in about 30 to 120 minutes, though the drift not reaching a steady state does not hinder measurements (logbook entries just need to be made more often, to accurately estimate the force).

(f) Return from alignment station

(g) Make a logbook entry.

Note: Now, it is important not to touch any of the knobs on the scan head, even if the signal keeps deviating away from 0,0 volts – we should be able to correct for the drift.

6. Cantilever tune

(a) With the laser aligned, click on “Cantilever Tune”.

(b) In the tuning window that pops up, we won’t be making use of the “Auto Tune” function.

(c) Right click on the white space in the settings column on the right and select “Show all”.

(d) The goal here is to search for a torsional resonance, and confirm that it is not a spurious vertical resonance coupling into the lateral channel (see the section on Coupling Check below) and then determine the ratio of response to drive amplitude.

(e) Copy all settings as shown in Fig.S8.

(f) Set the two plots to auto scale by right clicking in the plot area and selecting “Auto scale”.

(g) Search for resonances, with a 1500 kHz center and a 3000 kHz width. At least two should show up, with a drive of 10 mV and 0.211 kHz bandwidth on the lock-in amplifier. If needed, save all spectra by clicking “Save Curve”.

(h) The resonance spectra will look similar to plots shown in Fig. 1(D) & (E) of the main text and Fig.S1.

(i) The resonance with the tallest peak can then be selected and a frequency window of about 500 kHz can set with the resonance at the center.

(j) Coupling check:

   i. Next, set “Coupling check” to “On”.

   ii. This mode replaces the displayed lateral amplitude with vertical amplitude, while continuing to drive the torsional resonance.

   iii. A purely torsional or lateral signal should not appear on the vertical deflection channel, with coupling check turned on.

   iv. If the resonance peak chosen appeared due to crosstalk from a vertical resonance, the previously observed peak or a shoulder of it (from the lateral channel) should become stronger. In such a case, this peak should not be chosen for imaging. If many such coupled peaks appear often, a different probe holder or different mounting of AFM tips must be tested.

   v. Next, set turn off coupling check, as imaging will be performed with coupling check turned off.

(k) Balance tune.

   i. Next, reduce the frequency window to between 50 kHz to 5 kHz with the resonance at the center.

   ii. Click “More” at the bottom of the screen and select “Balance Tune”.

   iii. The instrument will automatically select the maxima of resonance after this.

   iv. Ideally, 0V and 10V refer to driving either the left or the right piezos with 5V on balance indicating both the piezos are being driven equally. An ideal placement of an AFM tip should lead to values around 5V on balance tune showing a maxima in resonance.

Note: Software bug: As of this writing, a bug in the software limits the usability of this feature and a local maxima is observed at either 0 or 10V of balance tune, which is selected.

v. Zero the phase by clicking "Zero phase".

(l) In "Sweep", set "TR drive amplitude" to 2 mV. A clear resonance at low drive voltages has been found to aid in imaging atomic lattices.

(m) Exit cantilever tune.

Note: Since this is the first instance after aligning the laser where significant time has elapsed. The vertical deflection value should have drifted from the previously set 0V to a few hundred millivolts. The sign of this drift and the magnitude are both indicators of the drift that would have to be corrected when imaging.

(n) Make a logbook entry.

7. Navigate to sample.

(a) Find and focus on the sample, but aim to land on a region that is not critical as there is always a risk of the AFM tip damaging the surface if approach parameters are not chosen correctly. For a tBG/hBN open face structure, making initial contact on hBN is better than making initial contact on tBG.

(b) Make a logbook entry.

8. Check parameters:

(a) From the settings shown in Fig.S9, copy all the settings from the "Scan" and "Feedback" categories.

(b) In "Torsion", the value for "TR Deflection Setpoint" will determine the force after contact and during imaging. It should be set to about 50 nN with respect to the "current" vertical deflection value. For example, if the vertical deflection is -400 mV (negative) and the AFM tip applies 1 nN for every 6 mV of signal, applying 50 nN (+300 mV)

Torsional Resonance - Cantilever Tune Settings

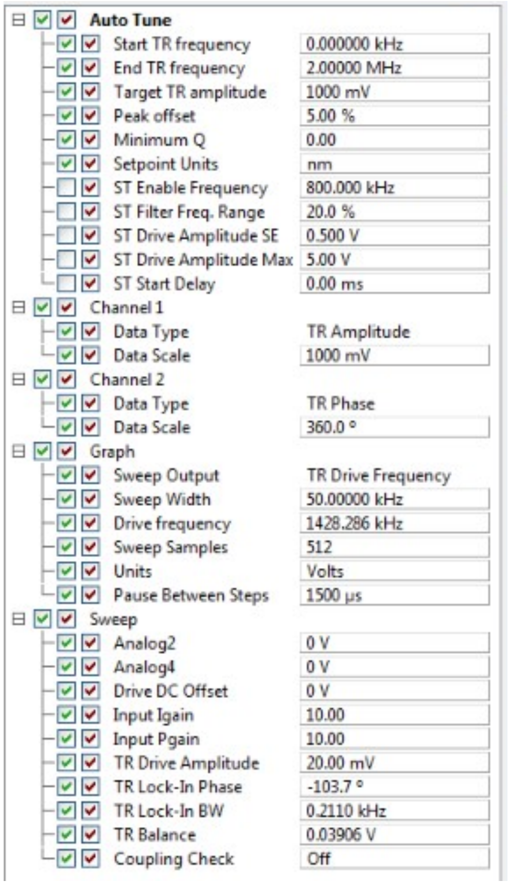

Fig. S8. Cantilever tune settings The cantilever tune settings are shown here as an example and may vary between cantilevers, probe holders and AFMs. Manual tune was used to first identify resonances in the torsional frequency spectrum and then using coupling check, were confirmed not to originate from crosstalk with vertical bending modes. Then with a finer frequency sweep centered at the resonance, the piezo balance was tuned and torsional drive amplitude for initial approach was set.

requires that -100 mV be entered in the TR Deflection Setpoint. Forces less than 50 nN may also work depending on the AFM tips used.

(c) Make a logbook entry.

(d) All the other settings in the “Torsion” category should have been carried over from cantilever tune, though the lock-in amplifier band width can be increased to 1 kHz to enable fast imaging initially.

(e) In the “Other” category, ensure the tip bias control and sample bias control are both set to “Ground”.

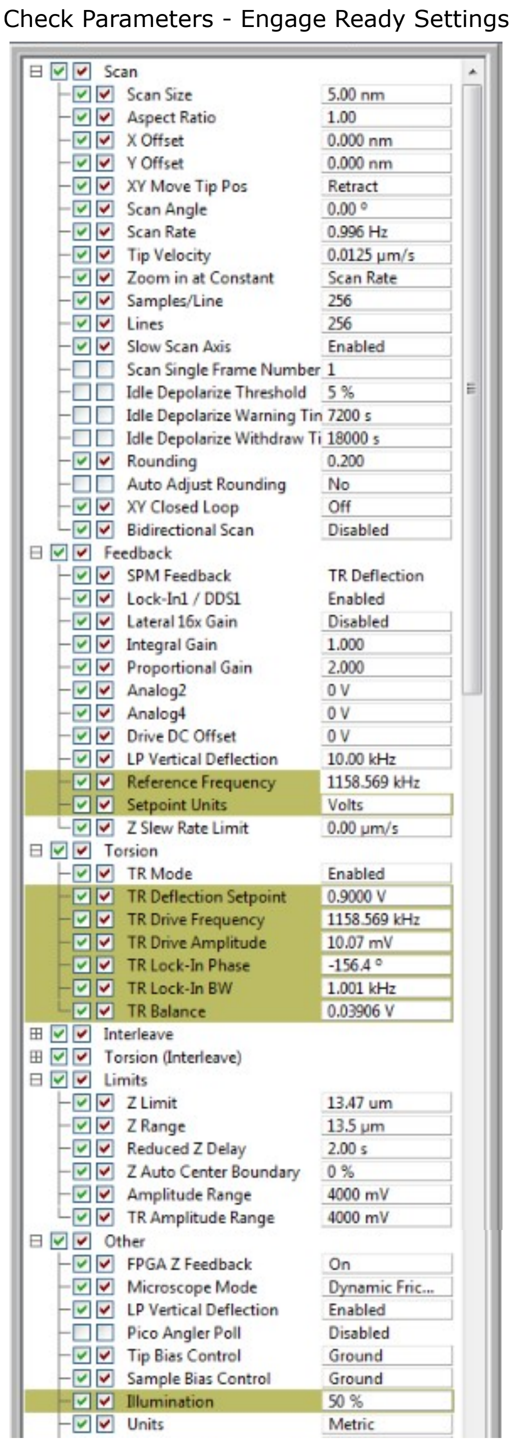

Fig. S9. Check parameters - Engage Ready Settings An example of the check parameters window is shown, immediately prior to engaging with the sample. A few key checks include confirming the “current” vertical deflection value and appropriately choosing the “TR Deflection Setpoint”, in volts, to make contact at about a nominal force of 50 nN.

9. Save the experiment and these workspace settings (to save time in future runs):
   (a) From check parameters window, go back to navigate and then to setup, to see the cantilever again.
   (b) Make a logbook entry.
   (c) Then from the Experiment menu at the top of the screen, save the experiment. This should save two files: experimentname.wks and experimentname.bag and enable recalling all the settings configured until this point the next time the instrument is turned on and this experiment started. Remember the location this experiment is saved to start from here.
   (d) Next, click on navigate to confirm if the region of interest is where to approach on the sample, and then click on check parameters to ensure a roughly 50 nN force is still what has been set by the TR Deflection Setpoint and the current value of vertical deflection.

10. Engage.
   (a) With the chosen force setpoint, engage the AFM tip with the sample.

   Note: It should take about a minute or two to engage. If the AFM tip is about 15 μm tall, the contact should be made with the sample at about 85 μm indicated by the current position indicator at the bottom of the screen.

   Note: Keep an eye on the vertical deflection setpoint during engage. If it shifts dramatically as the tip nears the sample but while its still not in contact, it indicates an electrostatic repulsion or attraction. Approach settings would then have to be tweaked accordingly.

A.4. Imaging with TFM:.

1. Setting up for imaging.
   (a) After a successful contact has been made, the instrument will start scanning the small scan region chosen entered previously.
   (b) The channels to be imaged would be "Height Sensor", "TR Deflection Error", "TR Amplitude", "TR Phase", "X Sensor", "Y Sensor". All channels should have no "OL" or Off-Line plane fitting, and the "RT" or Real-Time plane fitting set to "Line". It may be beneficial to record all of the above channels for either trace or retrace while the remaining two channels can be TR Amplitude and TR Phase for the opposite scan direction.
   (c) Record movie continuously or "forced" should also be enabled so that the data can now be saved.
   (d) To scan larger areas, increasing the P & I settings may be required, depending upon the undulations in the sample. Values of the order of 4 (I) and 8 (P) should enable imaging over tens of microns of scan areas with 100s of nm undulation, but these may vary from instrument to instrument.
   (e) Next, we increase the scan area to find the region of interest (R.O.I.). This initial region of interest can be something close to the most critical R.O.I. as there is another round of optimization of force and drive frequency before we can image the most critical R.O.I.

   Note: From the main text, "Typical line scan speeds (each line consisting of both trace and retrace) ranged from 2 Hz over microns, to 4 Hz over hundreds of nanometers and 30 Hz over tens of nanometers. At these speeds, the lock-in amplifier input bandwidth was typically set between the lower end of 0.211 kHz (limited by electronics) to 10 kHz, with increasing bandwidth at increasing speeds, to avoid digitization.". Adjust the scan speed and bandwidth accordingly.

   (f) Once the R.O.I. has been identified, reduce the scan area down to about 100 nm.
   (g) Then reduce the Z limit to about 4 microns. This should limit the Z range also. This option may not be available in newer versions of software and hardware.
   (h) Next, determine "Contact + 0 nN":
      i. Reduce the "TR Deflection Setpoint", by 10 nN approximately. i.e. if the setpoint where the sample was successfully approached and the scanning is stable was -0.1 V, make it -0.15V or -0.2V. Observe the voltage on the Z piezo.
      ii. At one such value the voltage on the Z piezo will become very negative, with the bar turning from green to yellow then red, indicating the piezo has retracted and is not tracking the surface anymore.
      iii. The goal is to determine within 10 mV the deflection setpoint at which the piezo retracts. For this, increase the setpoint again by about 0.2V to put the AFM tip back in contact and repeat the process, but with more finer steps as the "flying condition" is nearing.
      iv. Make a logbook entry of the flying condition and the last point of contact.

v. Then slowly increase the force in 10 mV steps to put the tip firmly back in contact, at which point the Z piezo will remain centered in the green region and the Z voltage value shown next to it will not change as the setpoint is increased. When the AFM tip first snaps back in contact, the deflection setpoint can be logged under the "Snapback" column. This value is more for information and troubleshooting and would not be necessary for imaging.
vi. Make a logbook entry.
vii. Next, with the "Contact + 0 nN" force determined, set the value to either 10 or 20 nN, by setting the appropriate deflection setpoint.
viii. This process of determining "Contact + 0 nN" should be repeated every 30 minutes if the vertical drift is found to be large or every 60-120 minutes if its small. After the first few hours or imaging, the instrument should reach a steady state where this drift should become negligible over hours.
ix. In the remaining text, if determine "Contact + 0nN" is called, repeat the above sequence again.
x. Every time an imaging surface is changed, i.e. for example going from tBG to hBN and vice versa, this process must be repeated as tip-sample interaction can dramatically alter the force applied for nominally the same values of "TR Deflection Setpoint".

(i) Next, determine the peak of torsional resonance:

i. During contact, due to interactions with the sample, the resonant frequency of the torsional resonance may have shifted (typically to more positive values).
ii. Open the "Generic Sweep" window by click on the button on the left.
iii. In the window that pops up, enter 0 nm. This will ensure the AFM tip remains in contact as the sweeps are being taken.
iv. The sweep window is similar to the cantilever tune window, but now with the auto tune function removed.
v. The goal is to plot the TR Amplitude in Channel 1 and TR Phase in Channel 2 (set both scales to auto scale) and find the torsional resonance peak.
vi. If a chosen peak doesn't yield desired results, other peaks (which are confirmed to be not present in the vertical deflection channel, using coupling check) can be chosen.
vii. After contact, the torsional resonance amplitude typical reduces from that in air, and the resonance frequency also shifts to a higher value. To find the peak, if it is not immediately apparent, increase the torsional drive amplitude to 10 or 20 mV and set the sweep width in the "Graph" category to about 100 kHz. A peak shift of about 1 kHz may not be surprising. The shifts will greater when the force is ramped up above 100 nN to search for moiré superlattices.
viii. Once the peak has been found, select "Center peak" to center it and "Zero phase". If auto centering doesn't work, use the offset and execute buttons to manually select the peak.
ix. Resonances with measured amplitudes between 10 to 50 mV would be sufficient for imaging, though optimal conditions may vary between instruments.
x. The impact of lock-in bandwidth can also be tested here. With the bandwidth set to 0.211 kHz, the noise on the resonance spectrum should be negligible. As bandwidth is ramped up, noise in both amplitude and phase should increase.
xi. A bandwidth of 1 kHz may be best suited for initial imaging and can be optimized later.
xii. Every time an imaging surface is changed, for optimal imaging, for example going from tBG to hBN and vice versa, this process must be repeated as tip-sample interaction can shift the torsional resonance.
xiii. Return to scan window by clicking "Exit".
Note: Software bug: As of this writing, exiting out of the generic sweep window in the torsional resonance mode resets the lock-in amplifier bandwidth to an arbitrarily high value (between 80 to 200 kHz). This value must be immediately, manually, set to the desired values in the "Torsion" category under "TR Lock-in BW" field. This bug will reset the bandwidth almost every time the generic sweep window is opened and some parameters tweaked.

2. Imaging moiré superlattices

(a) An example of imaging conditions for moiré superlattices is shown in Fig.S10(A).
(b) With the above complete, move into the R.O.I. where a moiré superlattice is expected.
(c) With the force set to Contact + 10 nN or 20 nN, image over the region of interest and zoom into the about 100 or 200 nm square. The scan speed, points per line and lock-in bandwidth can all now be increased.
(d) Then, lower the force to Contact + 0 nN and image the region.
(e) Step up the force in steps of 10 nN until a moiré appears – in either amplitude or phase or both. For example, if the most recent "Last point of contact" was -0.1 V, then increase the force to -0.04V, +0.02V, +0.08V and so on. Sudden increases in force may damage both the AFM tip and sample as this is essentially a contact AFM technique.

(f) Once the moiré contrast is optimized at a set force with a set torsional drive amplitude, open the sweep window again and tune to the peak of torsional resonance as it may have shifted with the increased force.

(g) Then, step through torsional drive amplitudes to optimize contrast further. Starting from about 2.5 mV, increase the drive amplitude in steps of 2.5 mV and observe the changes to the moiré superlattice. A sign that the drive amplitude is too high is when sharp features of the superlattice become broadened. The lowest torsional drive that yields the desired results is preferred. Depending on the AFM cantilever, high torsional drive voltages may be needed. Due limitations of DACs, the system can only output in steps of 0.3 mV. Finer steps would not affect the output to the piezos.

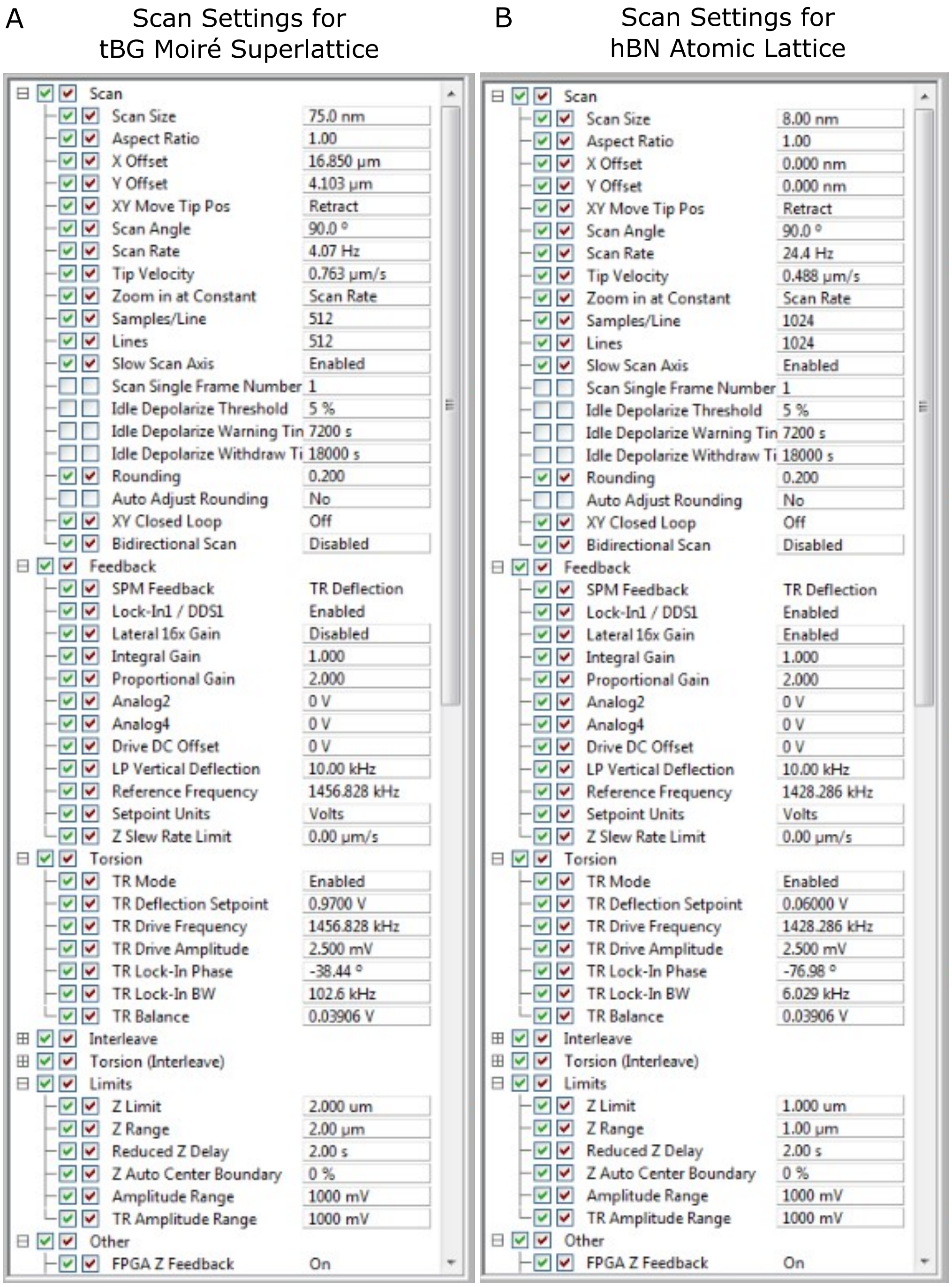

Fig. S10. Imaging settings for moiré superlattices and atomic lattices (A) An example of settings to image a tBG moiré and (B) the atomic lattice of hBN are shown with the key parameters being force, shown by TR Deflection Setpoint represented in volts, torsional drive amplitude, shown by TR Drive Amplitude, in millivolts. The lock-in bandwidth for (A) is over 100 kHz as force and drive amplitude were changed to find the optimal settings for imaging and was reduced once ideal settings were found. Note the larger area image in (A) is taken at a line scan speed of 4.07 Hz while the relatively smaller image in (B) is taken at 24.4 Hz.

(h) Once both optimal conditions for force and torsional drive amplitude have been determined, open the frequency sweep again to confirm the resonance peak is selected. Then after returning from the sweep window, reduce the bandwidth to the most optimal.

(i) The above protocol was used in main text Fig. 3 and has been shown. Now the scan area can be changed from many microns to many nanometers without changing anything more than scan speed and with it the bandwidth. The torsional drive amplitude and force do not have to be changed, for this region.

(j) Some additional optimizations are mentioned after the note on imaging atomic lattices.

3. Imaging atomic lattices.

(a) An example of imaging conditions for atomic lattices is shown in Fig.S10(B).

(b) With the above complete, move into the R.O.I. where the atomic lattice has to be imaged.

(c) With force set to about contact + 10 nN or contact + 20 nN, image over the region of interest and zoom into the about 5 to or 20 nm square. The scan speed, points per line and lock-in bandwidth can all now be increased.

(d) Then, lower the force to Contact + 0 nN and image the region.

(e) Line scan speeds of 8-30 Hz were often used in conjunction with the lowest bandwidth, for that line scan speed, that did not show any signs of digitization in the line profile.

(f) Points per line was increased to 512x512 typically.

(g) Step up the force in steps of 10 nN, with torsional drive amplitude set to between 2-5 mV, until an atomic lattice appears – in either amplitude or phase or both. For example, if the most recent "Last point of contact" was -0.1 V, then increase the force to -0.04V, +0.02V, +0.08V and so on. Sudden increases in force may damage both the AFM tip and sample as this is essentially a contact AFM technique. Ideal contrast should not require forces much greater than 50 nN for surfaces of hBN. Other materials may behave differently. VdW flake thickness may play another critical role and thinner flakes may show poorer contrast.

(h) Once the atomic lattice appears, open the sweep window again and tune to the peak of torsional resonance, again as it may have shifted with the increased force.

(i) Then, step through torsional drive amplitudes to further optimize contrast. Starting from about 1 mV, increase the drive amplitude in steps of 1 mV and observe the changes to the atomic lattice. The lowest torsional drive that yields the desired results is preferred. Depending on the AFM cantilever, high torsional drive voltages may be needed. Due limitations of DACs, the system can only output in steps of 0.3 mV. Finer steps would not affect the output to the piezos.

(j) Once both optimal conditions for force and torsional drive amplitude have been determined, open the frequency sweep again to confirm the resonance peak is selected. Then after returning from the sweep window, reduce the bandwidth to the most optimal.

(k) The above protocol was used in main text Fig. 2(B). Now the scan area can be changed from about 3 nm to 20 nm to while keeping the speed and bandwidth the same to confirm if the image represents and atomic lattice and not periodic noise. The torsional drive amplitude and force do not have to be changed.

(l) Some additional optimizations are mentioned below.

4. Some common suggestions applicable to both moiré superlattice and atomic lattice imaging.

(a) Lower the gain to value like 1(I) and 2(P), when imaging over a smooth surface. If the gain is high, ringing would be apparent in the TR Deflection Error channel.

(b) The scan angle can be changed from 0° to 90° or other values.

(c) The parameter of "Rounding" set to 0.2 for scans about 100nm or smaller in open loop X-Y mode enables scanning 10% excess on either side of the fast scan axis and may helps remove artifact at the edges of the scan frame.

(d) To optimize imaging, the "Lateral 16x gain" from the feedback category can be enabled. This only amplifies the TR amplitude and not the phase.

(e) Alternatively, if the signal is fairly large already (over 100 mV), then the "TR Amplitude Range" and "Amplitude Range" can be reduced from 4000 mV to a lower value. At a range of 4000 mV, the lock-in is configured to take in an input of ± 2000 mV. For a 100 mV signal if 16x gain is turned on, that makes the signal 1600 mV and hence the full 4000 mV range of lock-in would be required. Conversely, if the signals are lower, the lock-in range can be reduced to increase sensitivity.

(f) Remember to save the data!

(g) Determine "Contact + 0 nN" every 30-120 minutes to ensure only the desired force is being applied. If the drift is making the vertical deflection shift to a more negative value, the force during imaging, is unintentionally and uncontrollably increasing (even though the feedback loop is operational to maintain the deflection setpoint) and could damage the sample.

(h) If both trace and retrace are being saved, the height sensor and TR deflection error channels can now be used to record the X-Y sensor data for the other pair of TR amplitude and phase.

5. Ending the imaging session.

(a) When the image session is coming to an end, it is important to record certain parameters so that the instrument drift (and hence drift in the force applied) can be quantified.

(b) Upon clicking "Withdraw", make a logbook entry.

(c) Move back to check parameters and make another logbook entry. Note the presence of the sample right below the AFM tip could affect the values measured and logging these values now would aid in determining the same.

(d) Next, move to navigate and move the sample to loading position.

(e) Once the sample is not under the tip anymore, move to setup and make a logbook entry. Any change in value since the last recorded value is due to the presence of the sample in the vicinity. The change from when the laser was first aligned to this final logbook entry, tells the extent of drift in the force, if purely nominal force values were chosen by entering deflection in nanometers with respect to the original setpoint of 0,0V. We found that forces in excess of 100 nN can be applied, unintentionally over a course of a few hours, if not logged accurately.

Note: The experiment can again be saved, but should be saved with a different name as the conditions saved would now be for imaging and not for instrument initialization. Initializing the instrument with these settings where the Z range may have been lowered, could be dangerous.

B. Image analysis (optional).

1. Images presented in this work were analyzed in Gwyddion.

Note: Software bug: As of this writing, when .spm files from TFM (torsional resonance mode of the instrument) are opened in Gwyddion, the Z axis values are changed from indicating mV for amplitude and degrees (°) for phase to showing volts (V). By analyzing the Z-scale of the data in NanoScope Analysis version 3.0, we confirmed that only the displayed units of volts are incorrect and can be swapped with mV and degrees in postprocessing.

2. The data was first corrected using the align rows function followed by fixing zero to the bottom of the scale.

3. Atomic lattice images could best be represented in an adaptive color scale (due to their relatively low contrast) while moiré superlattice images could be represented in all color scales (typically the linear scale was chosen).

4. 2D FFT analysis was also performed using the in-built functions to measure the lattice.

5. X-Y sensor-based postprocessing to correct for scanner piezo creep and hysteresis was not employed in this work. Coupled with thermal drift, piezo creep and hysteresis introduces uncertainty in precise determination of moiré period, twist angle and strain as well as angular orientation of atomic lattices.

C. Estimating torsional deflection sensitivity (in units of pm/mV) and amplitude of spatial torsional deflection (in units of pm, peak to peak) (optional).

Note: This sequence of steps assumes the reader will rely on the provided Jupyter notebook for estimation of torsional deflection sensitivity and torsional deflection amplitude. These steps have also been provided in the Jupyter notebook for ease of use.

1. Capture optical microscope images of the cantilever to be used.

Note: If the tip height h and thickness of the cantilever t can be imaged with a side view or are available from SEM, then the measured h and t should be used.

2. Install the AFM tip in any holder (incl. the torsional probe holder). Align the laser as normal for imaging. For direct correlation with TFM results, use a torsional probe holder and perform all of the remaining steps in one sitting, i.e. without readjusting the laser spot on the AFM cantilever between calibration and TFM imaging.

3. From any imaging mode of the NanoScope software, switch the microscope mode to "Contact" and turn off all lock-in amplifiers in the software. Also disable the LP Friction and LP Deflection low pass filters. This should, in principle, turn off all oscillators in the system. An example of the workspace settings file to ensure all oscillators have been turned off is included in the raw data.

4. Open HSDC capture to capture high speed data. Acquire vertical deflection and lateral deflection channel signals using the 6.25 MHz acquisition rate, for the longest allowed time (about 2.5 seconds). Save the generated .hsdc data file in a convenient location. An example .hsdc file has been included in the raw data.

5. Using thermal tune, carefully record the resonant frequency of the first vertical bending mode (not the torsional resonance frequency).

6. Import the .hsdc data in the provided Jupyter notebook (python version 3.11) and enter the cantilever dimensions and its vertical resonant frequency. In the same notebook, select the appropriate frequency range where the torsional resonance is expected (which should be the same resonance used for TFM imaging).

7. Complete any additional steps as may be mentioned in the Jupyter notebook and compute the torsional deflection sensitivity using the notebook.

8. (Optional) Image using TFM. Note the resonance frequency for imaging as well as the torsional resonance spectra used for imaging (specifically the peak torsional resonance amplitude in mV) at a set vertical loading force.

9. (Optional) From the peak of the torsional resonance measured (in mV) above, for a particular vertical loading force and imaging conditions, approximate the peak-to-peak amplitude of deflection of the tip apex, using the Jupyter notebook.

References

1. AL Eichhorn, C Dietz, Torsional and lateral eigenmode oscillations for atomic resolution imaging of HOPG in air under ambient conditions. *Sci. Reports* **12**, 8981 (2022).
2. C Su, L Huang, CB Prater, B Bhushan, Torsional Resonance Microscopy and Its Applications in *Applied Scanning Probe Methods V: Scanning Probe Microscopy Techniques*, NanoScience and Technology, eds. B Bhushan, S Kawata, H Fuchs. (Springer, Berlin, Heidelberg), pp. 113–148 (2007).
3. Bruker Corp. TR mode Support Note 416 (Rev. I) (2011).
4. N Mullin, JK Hobbs, A non-contact, thermal noise based method for the calibration of lateral deflection sensitivity in atomic force microscopy. *Rev. Sci. Instruments* **85**, 113703 (2014).
5. M Pendharkar, et al., Data for: Torsional Force Microscopy of Van der Waals Moires and Atomic Lattices (2023) Stanford Digital Repository. https://doi.org/10.25740/zj475qr8207.
